# Dual‐Channel Fluorescence Assays with Supramolecular Host‐Dye Reporter Pairs for Membrane Activity Mapping of Peptides

**DOI:** 10.1002/anie.202517709

**Published:** 2025-12-13

**Authors:** Mohammad A. Alnajjar, Sandra N. Schöpper, Malavika Pramod, Thomas Reingolz, Lina Müller, Justin Neumann, Mohamed Nilam, Werner M. Nau, Andreas Hennig

**Affiliations:** ^1^ Center for Cellular Nanoanalytics (CellNanOs) and School of Biology / Chemistry Universität Osnabrück Barbarastraße 7 49069 Osnabrück Germany; ^2^ School of Science Constructor University Campus Ring 1 28759 Bremen Germany

**Keywords:** Cell‐penetrating peptides, Fluorescence assays, Membrane pores, Permeability, Supramolecular chemistry

## Abstract

Membrane‐active peptides (MAPs) are a major class of peptides that renders lipid bilayer membranes permeable for hydrophilic compounds. MAPs include cell‐penetrating peptides (CPPs) and pore‐forming antimicrobial peptides (AMPs), which are believed to be mechanistically related. CPPs render the membrane sufficiently permeable to enable their own translocation, while AMPs create membrane damage and induce cell death. We report herein a fluorescence‐based, dual‐channel assay, which combines a classical dye efflux assay based on self‐quenched carboxyfluorescein (CF) and a recently established supramolecular tandem membrane assay based on the supramolecular host‐dye complex of *p*‐sulfonatocalix[4]arene (CX4) and lucigenin (LCG). The new assay provides a functional classification of MAPs, which distinguishes between their capability to directly translocate across the vesicle membrane or to induce sufficient membrane permeability to allow dye efflux. The assay was validated with melittin, penetratin, Pep‐1, TP10, and various oligoarginine peptides including the TAT peptide, which confirmed their classification as CPPs or pore‐forming peptides. An advanced variant of the tandem membrane assay also allowed to distinguish between the formation of transient pores and stable equilibrium pores. Overall, the established dual‐channel assay provides a simple and easy to implement method for the advanced mechanistic characterization of MAPs and an exploration of their mechanistic landscape.

## Graphical abstract

Membrane‐active peptides (MAPs) such as cell‐penetrating peptides (CPPs) and antimicrobial peptides (AMPs) can translocate across lipid membranes and create membrane pores. The combination of a dye efflux and a supramolecular tandem membrane assay in a dual‐channel format enables an advanced mechanistic characterization and an exploration of the mechanistic landscape of MAPs enabling their straightforward functional classification.

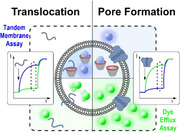

## Introduction

Peptides and peptide‐inspired molecules are receiving significant interest as biochemical research tools and as therapeutic agents.^[^ ^1^, ^2^, ^3^ ^]^ This includes cell‐penetrating peptides (CPPs), which are useful as cellular delivery vectors for membrane‐impermeable cargoes,^[^ ^4^, ^5^, ^6^ ^]^ and pore‐forming antimicrobial peptides (AMPs), which are urgently needed as last resort antibiotics.^[^ ^7^, ^8^, ^9^, ^10^, ^11^ ^]^ The mechanisms of these peptides have been intensively explored and largely debated, which has led to the current hypothesis that these peptides are mechanistically related.^[^ ^12^, ^13^ ^]^ Since most representatives of these peptide classes can permeabilize lipid bilayer membranes, yet to different extents, it is currently believed that these peptides operate on a mechanistic continuum (or landscape).^[^ ^14^, ^15^, ^16^ ^]^ An ideal AMP leads to effective and irreparable membrane damage and ultimately cell death, whereas an ideal CPP renders the membrane just permeable enough to locally translocate the CPP (and its conjugated cargo) without compromising cellular integrity. Jointly, these peptides can be categorized as membrane‐active peptides (MAPs).

The biophysical characterization of MAPs includes structural characterization methods, such as NMR and circular dichroism spectroscopy, imaging techniques like fluorescence microscopy with cells and giant unilamellar vesicles (GUVs), computational methods such as molecular dynamics simulations, as well as synthesis‐driven structure–activity relationship studies.^[^ ^17^, ^18^ ^]^ However, there is an imminent need for high‐throughput screening (HTS) approaches to aid in the discovery and characterization of membrane‐active peptides.^[^ ^19^, ^20^, ^21^, ^22^ ^]^ Fluorescence spectroscopic assays are a classical research method to characterize membrane‐active compounds^[^ ^23^, ^24^, ^25^, ^26^, ^27^, ^28^, ^29^ ^]^ and they have a demonstrated potential for HTS^[^ ^30^, ^31^, ^32^, ^33^ ^]^ with the possibility for single‐molecule detection.^[^ ^34^, ^35^, ^36^ ^]^

**Scheme 1:**
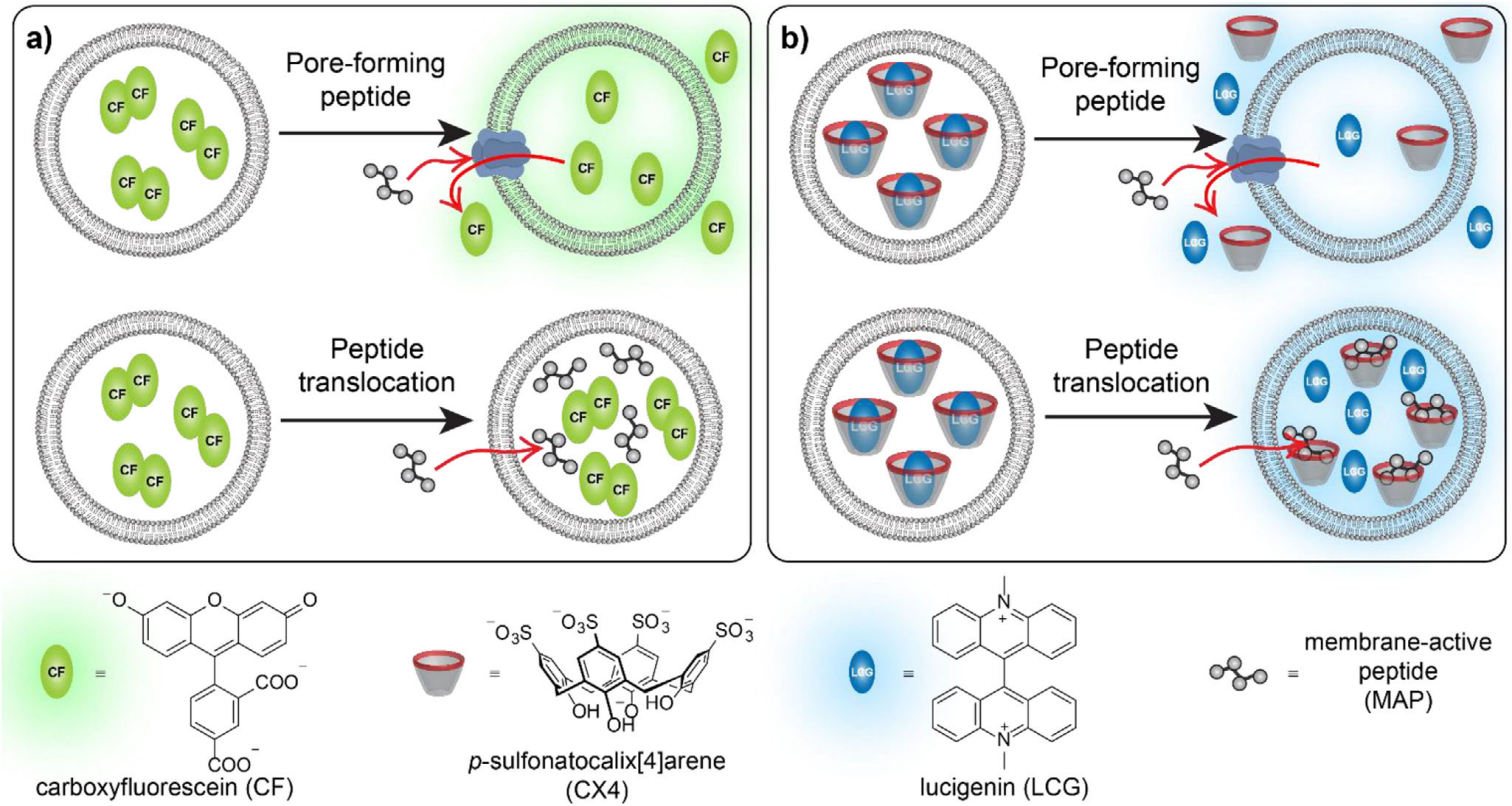
a) Principle of a dye efflux assay. The fluorescent dye 5(6)‐carboxyfluorescein (CF) is encapsulated at self‐quenching concentrations in large unilamellar vesicles (LUVs). Dye efflux leads to dilution and a fluorescence increase. b) Principle of supramolecular tandem membrane assay. The fluorescent dye lucigenin (LCG) and the macrocyclic host *p*‐sulfonatocalix[4]arene (CX4) form a host‐dye reporter pair that is encapsulated in LUVs. A fluorescence increase results either from efflux through a sufficiently large membrane pore or from intravesicular dye displacement after peptide translocation.

The most common fluorescence spectroscopic techniques for characterizing MAPs are dye efflux assays, which are also referred to as dye leakage assays (Scheme 1a). Therein, a fluorescent dye (or a dye/quencher pair) is encapsulated at high concentrations in the interior of large unilamellar vesicles (LUVs). The high intravesicular concentrations lead to efficient fluorescence quenching, while efflux or leakage from the vesicle interior leads to dilution of dye and/or quencher into the bulk solution and a concomitant fluorescence increase. Dye efflux assays have become extremely popular, because they are readily set up, do not require specialized instrumentation, and enable a straightforward determination of the activity of pore‐forming peptides. They are also established research tools in supramolecular chemistry for investigating and characterizing the membrane activity of artificial membrane pores.^[^ ^23^, ^24^, ^25^, ^26^, ^27^, ^28^, ^29^ ^]^ However, dye efflux assays are limited in their ability to characterize CPPs, because peptide translocation can be only indirectly monitored. Dye efflux assays require that the membrane becomes sufficiently permeable during the translocation step to enable the escape of the encapsulated dye (or quencher); consequently, some MAPs may evade detection. For example, spontaneous membrane‐translocating peptides (SMTPs), another variant of MAPs, have been termed silently translocating peptides, because they efficiently pass through lipid bilayer membranes but remain spectroscopically silent in conventional dye efflux assays.^[^ ^30^, ^37^, ^38^, ^39^, ^40^, ^41^, ^42^, ^43^, ^44^, ^45^ ^]^

We have recently introduced a method for real‐time fluorescence monitoring of membrane‐permeable and membrane‐active compounds, which we have termed tandem membrane assays (Scheme 1b).^[^ ^46^, ^47^, ^48^, ^49^, ^50^, ^51^ ^]^ The method uses supramolecular host‐dye reporter pairs, which are self‐assembled complexes of macrocyclic host molecules and fluorescent dyes, in which the dye is either quenched or its fluorescence is significantly increased in the complexed form. The membrane‐impermeable reporter pair is encapsulated in the lumen of LUVs and the compound of interest is subsequently added to the extravesicular solution. If the compound is membrane‐permeable, it can translocate across the vesicle membrane, bind to the macrocyclic host and thereby displace the fluorescent dye, which leads to a detectable change in fluorescence intensity. Tandem membrane assays differ from classical dye efflux assays, because they can be used to monitor passive diffusion of low‐molecular weight compounds across lipid bilayer membranes (i.e., membrane permeation),^[^ ^48^, ^49^, ^50^ ^]^ and they have also been used for real‐time fluorescence monitoring of peptide translocation.^[^ ^52^ ^]^ This becomes possible, because the intravesicular molecular recognition of the peptide by the macrocyclic host and the concomitant dye displacement affords a detectable spectroscopic output signal for otherwise silently translocating peptides (cf. Scheme 1a,b).

Herein, we evaluate a selection of MAPs including pore‐forming peptides, CPPs, and SMTPs with both assays – the CF dye efflux assay and the CX4/LCG supramolecular tandem membrane assay. The assays were, first, optimized to make the results from both assays directly comparable, and further developed into a dual‐channel assay format, which enabled monitoring of both assays in the same sample at the same time. The newly developed method revealed significantly higher membrane activities of CPPs with the CX4/LCG tandem membrane assay than with the CF dye efflux assay, which conclusively demonstrates that these peptides are more efficient in translocating across the vesicle membrane than in inducing leakage from vesicles, while the opposite behavior was found for pore‐forming MAPs. Overall, our method allows to mechanistically dissect peptide translocation from pore formation and conclusively confirms that MAPs indeed operate on a continuous mechanistic landscape.

## Results and Discussion

In order to cover the various major subclasses of MAPs, we have selected some famous prototypes of each class (Table 1). As a pore‐forming peptide, we chose the hemolytic bee venom melittin, which is an α‐helical peptide that forms disordered toroidal pores,^[^ ^32^, ^33^, ^53^ ^]^ and as SMTP, we included the LRLLRW peptide, which is a truncated, minimal version of the TP2 peptide.^[^ ^30^, ^37^, ^38^, ^39^ ^]^ CPPs include polycationic peptides such as penetratin and TAT_48‐60_, which are truncated versions of full‐length proteins and were the first two CPPs discovered.^[^ ^54^, ^55^, ^56^, ^57^ ^]^ Both inspired the development of oligoarginine CPPs such as heptaarginine (R7) and nonaarginine (R9),^[^ ^58^ ^]^ which we also included. TP10 is a hydrophobic CPP derived from transportan, which is a chimeric peptide of the neuropeptide galanin and the wasp venom mastoparan,^[^ ^59^, ^60^, ^61^, ^62^, ^63^ ^]^ and Pep‐1 (also known as Chariot peptide) is an amphipathic CPP composed of a hydrophobic, N‐terminal domain and a hydrophilic, lysine‐rich C‐terminal domain separated by a proline residue.^[^ ^64^, ^65^ ^]^

**Table 1:** Binding constants of peptides to CX4.^a)^

Peptide	Sequence	*K*_a_ (M^−1^)
Melittin	H‐Gly‐Ile‐Gly‐Ala‐Val‐Leu‐Lys‐Val‐Leu‐Thr‐Thr‐Gly‐Leu‐Pro‐Ala‐Leu‐Ile‐Ser‐Trp‐Ile‐Lys‐Arg‐Lys‐Arg‐Gln‐Gln‐NH_2_	(1.2 ± 0.3) × 10^7^
Penetratin	H‐Gln‐Ile‐Lys‐Ile‐Trp‐Phe‐Gln‐Asn‐Arg‐Arg‐Met‐Lys‐Trp‐Lys‐Lys‐OH	(9.9 ± 1.8) × 10^7^
TAT_48‐60_	H‐Gly‐Arg‐Lys‐Lys‐Arg‐Arg‐Gln‐Arg‐Arg‐Arg‐Pro‐Pro‐Gln‐OH	(1.9 ± 0.9) × 10^8^
R7^b)^	H‐Arg‐Arg‐Arg‐Arg‐Arg‐Arg‐Arg‐OH	(7.2 ± 0.5) × 10^7^
R9	H‐Arg‐Arg‐Arg‐Arg‐Arg‐Arg‐Arg‐Arg‐Arg‐OH	(1.8 ± 0.4) × 10^8^
Pep‐1	H‐Lys‐Asp‐Thr‐Trp‐Trp‐Asp‐Thr‐Trp‐Trp‐Thr‐Asp‐Trp‐Ser‐Gln‐Pro‐Lys‐Lys‐Lys‐Arg‐Lys‐Val‐OH	(2.8 ± 0.2) × 10^6^
TP10	H‐Ala‐Gly‐Tyr‐Leu‐Leu‐Gly‐Lys‐Ile‐Asn‐Leu‐Lys‐Ala‐Leu‐Ala‐Ala‐Leu‐Ala‐Lys‐Lys‐Ile‐Leu‐OH	(7.2 ± 0.9) × 10^5^
LRLLRW	H‐Leu‐Arg‐Leu‐Leu‐Arg‐Trp‐NH_2_	(1.3 ± 0.1) × 10^6^

^a)^ Measured by LCG displacement in 10 mM Hepes, pH 7.0.

^b)^ In 10 mM NaH_2_PO_4_, pH 7.2; taken from Ref. [46]. Errors correspond to the standard deviation obtained by nonlinear fitting (*n* = 1).

To test whether the different membrane‐active peptides bind to CX4 and thereby displace LCG to afford a fluorescence response in a tandem membrane assay, binding constants of the different peptides to CX4 were determined by competitive dye displacement using LCG (Figures S1–S8). This revealed nanomolar affinity of all peptides except for TP10 (Table 1). TP10 contains only few positively charged amino acid residues and binding is therefore comparably weak. The data reflect that binding of the polycationic peptides to negatively charged CX4 is mainly driven by electrostatic interactions rendering the combination of CX4 with LCG an excellent sensor for a large variety of MAPs.

**Figure 1:**
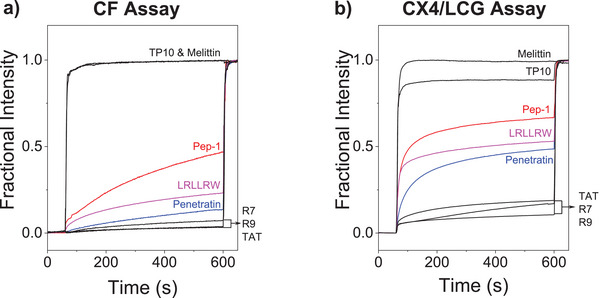
a) Time‐dependent changes in fractional intensity of EYPC⊃CF‐LUVs (*λ*_exc_ = 492 nm, *λ*_em_ = 517 nm) in 10 mM Hepes, 97 mM NaCl, pH 7.5, 25 °C, after addition of different peptides (30 µM at 60 s), and 20 µL 1.6% (wt/vol) TX‐100 at 600 s. b) Time‐dependent changes in fluorescence intensity of POPC⊃CX4/LCG‐LUVs (*λ*_exc_ = 369 nm, *λ*_em_ = 502 nm) in 10 mM Hepes, pH 7.0, after addition of different peptides (30 µM at 60 s) and 20 µL calibration cocktail (1.6% (wt/vol) TX‐100 and 1.5 mM protamine) at 600 s.

All peptides were subsequently screened at high concentrations with the CF dye efflux assay and the CX4/LCG tandem membrane assay (Figure 1). The CF assay revealed high membrane activities for the pore‐forming peptide melittin and no significant membrane activity for the cationic CPPs TAT_48‐60_, R7, and R9. These results are fully consistent with previous reports, in which melittin or oligoarginine peptides were added to CF‐loaded vesicles with neutral phospholipids.^[^ ^52^, ^66^, ^67^ ^]^ A high membrane activity in the CF assay was also observed for the CPP TP10, which is known to translocate across membranes by formation of sufficiently large pores through which dyes can escape.^[^ ^62^, ^63^ ^]^

A direct comparison between the CF assay and the CX4/LCG tandem membrane assay suggested, however, an interesting trend, namely that the membrane activity of nearly all CPPs was higher with the CX4/LCG assay compared to the CF assay (Figure 1 and Table S4). Since the initial screening was performed with different lipids and with different ionic strength, we next aimed to establish identical conditions for both assays to confirm that the subtle, yet consistent differences in the membrane activities of the CPPs do not originate from the different assay conditions.

To test for the influence of different lipids, the CPP Pep‐1 was selected, because it showed measurable activities in both assays. This allowed the determination of the respective *EC*_50_ values (i.e. the concentration required to reach 50% membrane activity) and enabled a quantitative comparison of membrane activities in egg yolk phosphatidylcholine (EYPC) and 1‐palmitoyl‐2‐oleoyl‐phosphatidylcholine (POPC) vesicles with both types of assays (Table S5 and Figures S10–S13). The results ruled out immediately that the higher activity with POPC⊃CX4/LCG‐LUVs compared to EYPC⊃CF‐LUVs (Figure 1) originated from the different lipids, because the membrane activity was consistently higher with EYPC lipids compared to POPC lipids regardless of the assay type (Table S5). Consistent results were obtained for penetratin (Figures S14 and S15), which suggested that the observed trend of a higher membrane activity of CPPs with the CX4/LCG assay compared to the CF assay is intrinsic to the assay type.

Next, potential influences of the different ionic strengths in the two assays needed to be ruled out. The different ionic strengths originate from the fact that the CF assay requires extravesicular NaCl to compensate for intravesicular CF at high, self‐quenching concentrations. This contrasts the CX4/LCG assay, in which NaCl is on purpose avoided, because Cl^–^ acts as quencher of LCG fluorescence (Figure S16). Consequently, an alternative osmolyte needed to be identified to establish identical conditions in the two assays; simple omission of NaCl in the CF assay was not possible and gave very fragile vesicles with >200‐fold lower *EC*_50_ values due to the osmotic stress caused by the hypoosmotic conditions (see Table S6 and Figures S17–S21).

As an alternative osmolyte, glucose was considered, which had been previously used for the investigation of MAPs with giant unilamellar vesicles (GUVs).^[^ ^63^, ^68^ ^]^ In the case of GUVs, glucose is added to the extravesicular medium and sucrose is added to the vesicle lumen to cause a desirable settling of the GUVs in fluorescence‐microscopic measurements. In our measurements with LUVs, we replaced extravesicular NaCl in the CF assay with 175 mM glucose to afford an isoosmolar solution that accounts for 50 mM intravesicular CF (and its respective counterion). To afford identical extravesicular conditions for both assays, the same amount of glucose was also added in the CX4/LCG assay, which had no significant influence on the CX4/LCG reporter pair (Figure S22).

**Figure 2:**
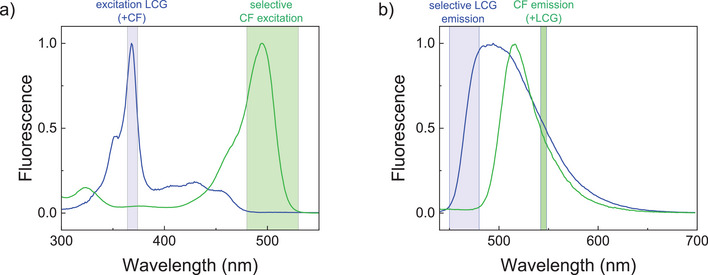
Normalized fluorescence a) excitation and b) emission spectra of CF (green) and LCG (blue). Selective excitation of CF is possible in the range from 480 to 550 nm (green box), while LCG emission can be selectively detected from 450 to 480 nm (blue box).

With the established identical extravesicular conditions in both assays, we further envisaged a simultaneous measurement of both assays within the same solution to enhance the comparability of the measurements. This became possible after optimization of the excitation and emission wavelengths. Inspection of the CF and LCG excitation and emission spectra suggested that CF can be selectively monitored by excitation above 480 nm where LCG does not absorb (Figure 2a), and that LCG can be selectively monitored in the range from 450 to 480 nm where CF does not emit (Figure 2b). A further optimization was required, because the dye concentrations in the CF assay and the CX4/LCG assay are largely different.^[^ ^51^, ^52^ ^]^ Consequently, simultaneous monitoring requires the detection of a low amount of LCG in the presence of a high concentration of the much more brightly fluorescent CF, which challenges the dynamic range of a typical fluorescence spectrometer in a dual‐channel measurement. To reduce the fluorescence read‐out of CF, the excitation wavelength was shifted from the absorption maximum at ca. 490 nm to 525 nm, where CF has a much lower absorbance, and the emission in the CF channel was shifted from its maximum at ca. 515 nm to 545 nm to further reduce the fluorescence of CF. To maintain a high LCG fluorescence, excitation was performed at the absorption maximum at 369 nm. Since CF has a residual absorbance at this excitation wavelength, emission in the LCG channel was monitored at 475 nm instead of 502 nm to eliminate spill‐over of CF fluorescence into the LCG channel.

During assay development and optimization, we also noted that protamine, which is added at the end of the CX4/LCG assay to ensure dissociation of the host‐dye reporter pair, influences CF fluorescence and thus tampers with the calibration of the CF assay. Presumably, anionic CF binds to the polycationic protamine leading to the formation of fluorescein dimers and concentration‐dependent fluorescence quenching (Figure S23).^[^ ^69^ ^]^ As an alternative, spermine was used, which is a tetracationic polyamine with nanomolar affinity to CX4 (Figure S24), but does not affect CF fluorescence (Figure S23b). With the optimized conditions, dual‐channel monitoring of CF‐LUVs, CX4/LCG‐LUVs, and both was performed during vesicle lysis, which confirmed that CX4/LCG‐LUVs and CF‐LUVs showed comparable fluorescence intensities (Figure S25). Moreover, spectral crosstalk was fully eliminated in the CF channel and negligible in the LCG channel (<10% of the overall fluorescence increase).

**Figure 3:**
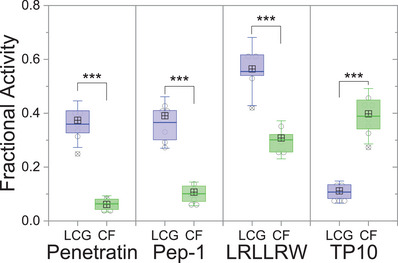
Results of unpaired, two‐tailed Student´s t‐test with penetratin (15 µM), Pep‐1 (10 µM), LRLLRW (30 µM), and TP10 (0.15 µM). The experimental data were obtained by dual‐channel measurements using seven independently prepared POPC⊃CX4/LCG‐LUV (*n* = 7) and POPC⊃CF‐LUV (*n* = 7) preparations diluted in 10 mM Hepes, 175 mM glucose, pH 7.5. Open circles (◯) represent individually measured data points, ⊞ is the median, and ⦻ are outliers. The boxes (⧮) refer to 25–75% distribution from mean (colored bar) ± 1.5 SD (whiskers). Probability values (*p*): * *p* value < 0.05, ** *p* < 0.01, *** *p* < 0.001.

Subsequently, *EC*_50_ values were determined for all peptides, which showed notable activity in our initial screening. These measurements were performed as single‐channel assays as well as in the newly developed dual‐channel mode (Figures S26–S39 and Tables S7–S9) and clearly confirmed the initially anticipated trend that the membrane activity of Pep‐1, penetratin, and LRLLRW was higher with the CX4/LCG assay compared to the CF assay. To confirm reproducibility of the results for different vesicle suspensions, seven independent batches of CX4/LCG‐ and CF‐LUVs were prepared, an appropriate peptide concentration was selected for each CPP based on its *EC*_50_ value, and the respective membrane activity was determined with a dual‐channel assay. Subsequently, a statistical analysis involving an unpaired Student's t‐test was performed (Figure 3).

The statistical analysis of the dual‐channel assay unambiguously revealed that the membrane activity of Pep‐1, penetratin, and LRLLRW was significantly higher (*n* = 7, *p* < 0.001) in the CX4/LCG channel than the membrane activity recorded in the CF channel. The result and its statistical significance was further confirmed by performing an analysis of variance (ANOVA), which also gave *p* < 0.001 for all four peptides (Table S10) indicating that the mean values for the two assays are different with >99.9% certainty for each peptide. This clearly suggests that these peptides have the capability to translocate across the vesicle membrane and displace LCG from the CX4/LCG reporter pair, while higher peptide concentrations are required to cause sufficiently large membrane perturbations through which the entrapped CF dye can escape. In theory, the signal change in the CX4/LCG assay requires stoichiometric amounts of translocated peptide to displace the LCG dye from CX4, whereas a single membrane pore principally should suffice to cause complete leakage of the entrapped dye in the CF assay. Consequently, the CX4/LCG assay rather underestimates the amount of peptide translocated in a direct comparison of the membrane activity values of the two assays, because a much smaller fraction of the peptide is sufficient to cause a signal change in the CF assay, whereas dye displacement in the CX4/LCG assay requires a substantial fraction of the peptide to be translocated. A similar trend, albeit with overall lower membrane activity, was also observed for the oligoarginine peptides R7 and R9 and for the TAT_48‐60_ peptide (Figure 1 and Figure S9).

Interestingly, TP10 was an exception among the CPPs and showed the opposite trend, namely a significantly higher membrane activity with the CF dye efflux assay compared to the CX4/LCG tandem membrane assay (Figure 3, right). The comparably high activity of TP10 in the CF dye efflux assay is consistent with the literature, which reports that TP10 translocates across membranes by formation of sufficiently large pores through which dyes can escape.^[^ ^60^, ^62^, ^63^ ^]^ The lower activity with the CX4/LCG assay readily excludes vesicle lysis and points toward a size exclusion effect of the membrane pores, in which the smaller CF passes more easily through the TP10 pores than the larger CX4 and LCG. The same behavior was also found for the pore‐forming peptide melittin, which also showed a higher activity with the CF assay compared to the CX4/LCG assay (Figure S35 and Table S9).

**Figure 4:**
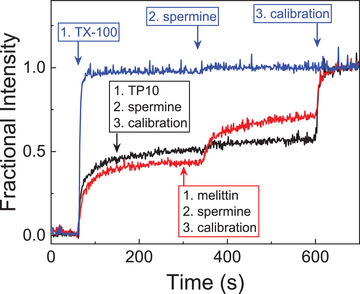
Changes in fractional intensity in the CX4/LCG assay showing the effect of addition of the strong competitor spermine (20 µM) after TP10 (0.2 µM, black) or melittin (5 nM, red) addition and incubation. The blue trace is a control to illustrate full dissociation of the CX4/LCG complex during vesicle lysis by TX‐100.

Interestingly, it has been reported that compound release by TP10 from vesicles proceeds via a graded mechanism.^[^ ^60^, ^70^ ^]^ This means that TP10 renders the vesicle membranes only transiently permeable, while after some time, the pores “close”, most likely due to equilibration of the inside and outside concentrations of TP10. At sufficiently low TP10 concentrations, the vesicles have consequently released only some of their content after a certain incubation time and no further release of encapsulated compounds is observed thereafter. The transient nature of the TP10 pores was also confirmed herein by the CX4/LCG tandem membrane assay (Figure 4, black line). After addition of TP10 at 60 s, the fluorescence increased for ca. 100 s and reached an intermediary plateau value, significantly below the value of vesicles that were lysed with TX‐100 (blue line). At this point of time, the TP10 pores should be either closed, or there should be some remaining stable equilibrium pores, which are too small to allow efflux of relatively large CX4 and/or LCG (MW > 500 Da), but sufficiently large to allow CF efflux (MW = 376 Da). Closed pores would refer to the previously reported graded mechanism, whereas the latter would point toward an all‐or‐none release mechanism.^[^ ^12^, ^70^ ^]^ The absence of a further fluorescence increase after addition of the even smaller spermine (MW ca. 200 Da) at 340 s (black line in Figure 4) clearly demonstrates the absence of stable TP10 pores and corroborates the graded release mechanism of TP10. The newly developed assay has therefore the added capacity to afford more fine‐grained mechanistic information on membrane activity.

The mechanism of pore formation of melittin in vesicles has been more controversial and includes reports of graded release from transient pores as well as reports of stable equilibrium pores.^[^ ^32^, ^33^, ^53^, ^66^, ^70^, ^71^, ^72^, ^73^ ^]^ When CX4/LCG vesicles were incubated with low concentrations of melittin, the increase in fractional fluorescence intensity reached a plateau after ca. 100 s incubation time. Subsequent addition of spermine at 340 s led to a further time‐dependent increase (red line in Figure 4), the magnitude of which was dependent on the concentration of added spermine (Figure S40). These results clearly indicate that melittin forms stable equilibrium pores, which are sufficiently large to enable influx of spermine, but too small to allow release of CX4 and/or LCG.

Ultimately, we have also tested the new conditions with vesicles containing the anionic lipid 1‐palmitoyl‐2‐oleoyl‐phosphatidylserine (POPS) with TP10, Pep‐1, and the LRLLRW peptide (Figures S41–S43 and Table S11). The results confirmed that the absolute activity as well as the difference between the CF dye efflux assay and the tandem membrane assay was nearly unaffected by the presence of 10% POPS with the only exception that the membrane‐translocating ability of TP10 was enhanced.

Overall, our results show that the combination of the CF efflux assay with a supramolecular tandem membrane assay provides mechanistic insights into the membrane activity of MAPs with unprecedented simplicity. The tandem assay alone reveals the membrane activity of otherwise silently translocating SMTPs,^[^ ^52^ ^]^ but only the combination of both assays under identical conditions enables to dissect the membrane‐translocating capability of CPPs from their ability to permeabilize membranes or to form pores. It is noteworthy that a fluorescence increase in the CF channel is not a definite proof for pore formation, but that other mechanisms may be operative, for example, a carrier‐type mechanism as suggested for counterion‐activated CPPs.^[^ ^67^ ^]^ Nonetheless, the dual‐channel assay clearly dissects peptide translocation into the vesicle from dye efflux out of the vesicles. The dual‐channel assay provides this result in a single measurement and additionally eliminates any uncertainties arising from incomplete mixing and membrane partitioning during peptide addition. In addition, the mechanistic differentiation between transient and stable equilibrium pores is very straightforward with the tandem membrane assay, while the prevailing method based on fluorescence re‐quenching^[^ ^60^ ^]^ requires the addition of high concentrations of quencher and several additional measurements at different quencher concentrations.

## Conclusion

In conclusion, we have optimized fluorescence‐based assays for the characterization of membrane‐active peptides (MAPs). Identical conditions for the CF dye efflux assay and the CX4/LCG supramolecular tandem membrane assay enabled for the first time an unambiguous comparison of the membrane activities by both assays as well as a simultaneous read‐out in dual‐channel mode. The assays were evaluated with pore‐forming peptides, CPPs, and SMTPs, which revealed significantly higher membrane activities of CPPs with the CX4/LCG tandem membrane assay compared to the CF dye efflux assay. This result is consistent with the ability of CPPs to directly translocate across the vesicle membrane without causing vesicle leakage that would be indicative of major damages to membrane integrity. Pore‐forming peptides showed the opposite trend, namely preferential leakage of the CF dye compared to the CX4/LCG reporter pair, which is consistent with a size exclusion effect of the formed membrane pores. The dual‐channel method thus allows the distinction between peptide translocation and pore formation in a simultaneous measurement with both assays. Advanced mechanistic investigations of MAPs become available by the addition of low‐molecular weight competitors for the CX4/LCG reporter pair after pre‐incubation with the MAPs, for example spermine, which allows to distinguish transient pores from stable equilibrium pores. Overall, the established dual‐channel assay provides a simple and easy to implement method for the advanced mechanistic characterization of MAPs and an exploration of their mechanistic landscape. Due to the fluorescence read‐out, high‐throughput measurement of membrane activity of extended peptide arrays could come into reach. At the present stage, a shortcoming is the incompatibility of the CX/LCG reporter pair with biological ionic strength, which calls for refined host‐dye reporter pairs.

## Supporting Information

The authors have cited additional references within the Supporting Information.^[^ ^74^, ^75^, ^76^, ^77^, ^78^ ^]^

## Conflict of Interests

The authors declare no conflict of interest.

## Supporting information



Supporting Information

## Data Availability

The data that support the findings of this study are available from the corresponding author upon reasonable request.
