# Supplementary material for: Dual‐Channel Fluorescence Assays with Supramolecular Host‐Dye Reporter Pairs for Membrane Activity Mapping of Peptides
Source: Angew Chem Int Ed Engl. 2025 Dec 13;65(5):e17709. doi: 10.1002/anie.202517709 (PMC12851007; doi:10.1002/anie.202517709)
Supplement: Supplementary file 1 — Supporting Information [file ANIE-65-e17709-s001.pdf]

Supporting Information

for

Dual-Channel Fluorescence Assays with Supramolecular Host-Dye Reporter Pairs for Membrane Activity Mapping of Peptides

Mohammad A. Alnajjar,[a] Sandra N. Schöpper,[a] Malavika Pramod,[a] Thomas Reingolz,[a] Lina Müller,[a] Justin Neumann,[a] Mohamed Nilam,[a] Werner M. Nau,*[b] Andreas Hennig*[a]

Table of Contents

Materials 2

Peptides 2

Other Materials 2

Instrumentation 2

Peptide Stock Solutions 2

Binding Constants 4

Membrane Activity Measurements 7

Vesicle Preparation 7

CX4/LCG-LUVs 7

CF-LUVs 8

Osmotically Stressed POPC⊃CF-LUVs 9

Dual-Channel Measurements 9

Activity Screening 10

Lipid Dependence 11

Pep-1: CF Assay and CX4/LCG Assay in POPC and EYPC 11

Penetratin: CX4/LCG Assay in POPC and EYPC 13

Influence of Osmotic Stress in the CF Assay 14

Chloride Quenching of LCG 14

POPC⊃CF-LUVs with and without extravesicular NaCl 14

Dual-Channel Assay Measurements 17

Influence of Glucose on CX4/LCG 17

Assay Development 17

CX4/LCG Assay with Glucose Buffer 19

CF Assay with Glucose Buffer 22

Dual-Channel Measurements 24

References 33

Materials

Peptides

Peptides were custom-synthesized by GL Biochem Ltd. (Shanghai, China) except for nonaarginine (R9), which was from NovoPro Bioscience Inc. (Shanghai, China), and heptaarginine (R7) and melittin, which were commercially available from Bachem AG (Bubendorf, Switzerland).

Other Materials

Lucigenin (LCG), 5(6)-carboxyfluorescein (CF), glucose, spermine, protamine sulfate (clupein, from herring), sodium chloride, Hepes, and Sephadex G-50 (BioReagent, for molecular biology, DNA grade, medium) were purchased from Sigma-Aldrich (Steinheim, Germany). Egg yolk phosphatidylcholine (EYPC) and 1-palmitoyl-2-oleoyl-*sn*-glycero-3-phosphocholine (POPC) were from Avanti Polar Lipids (Alabaster, AL, USA) and *p*-sulfonatocalix[4]arene sodium salt (CX4) was from TCI Deutschland. Deuterated solvents for NMR measurements were from Deutero (Kastellaun, Germany). Triton X-100 was from Alfa Aesar and sodium hydroxide was from Fluka.

Instrumentation

Ultrapure water was obtained from a Merck Millipore Simplicity® UV system. Fluorescence spectra and kinetic measurements were performed with a JASCO FP-8300 spectrofluorometer with temperature controller, stirrer, and pipette port. Absorption spectra were measured on a JASCO V-750 spectrophotometer with temperature controller. The size distribution of the liposomes was measured by dynamic light scattering (DLS) with a Malvern Instruments Zetasizer Nano. The osmolarity of the buffers was determined by a Vogel OM 807 osmometer. Extrusion of vesicle solutions was performed with an Avanti Lipid Extruder.

Peptide Stock Solutions

Peptide stock solutions were prepared in Millipore water at ca. 3 mM. The concentrations of the stock solutions were determined by measuring the absorbance at 214 nm and considering the extinction coefficients according to Table S1. The concentration of LRLLRW-NH_2 was determined using the extinction coefficient of Trp (ε_{280} = 5540 M^{-1} cm^{-1}).[1] Stock solutions of melittin were freshly diluted for individual membrane activity measurements and used immediately.

Table S1. Molar extinction coefficients of chromophoric groups in peptides.[a]

ε_{214} (M^{-1} cm^{-1})	Peptide						
	Penetratin	Tat	Arg9	Pep-1	TP10	Arg7	Melittin
peptide bonds	18 × 923	14 × 923	8 × 923	20 × 923	21 × 923	6 × 923	25 × 923
Arg side chains **R**	2 × 102	6 × 102	9 × 102	1 × 102		7 × 102	2 × 102
Phe side chain **F**	1 × 5200						
Gln side chains **Q**	2 × 142	2 × 142		1 × 142			2 × 142
Ile side chains **I**	2 × 45				2 × 45		3 × 45
Lys side chains **K**	4 × 41	2 × 41		5 × 41	4 × 41		3× 41
Trp side chains **W**	2 × 29050			5 × 29050			1 × 29050
Met side chains **M**	1 × 980						
Thr side chains **T**				3 ×41			2 ×41
Glu side chains **E**				3 × 78			
Ser side chains **S**				1× 34			1× 34
Pro side chains **P**		2 × 30		1 × 30			1 × 30
Val side chains **V**				1 × 43			2 × 43
Asn side chains **N**	1 × 136				1 × 136		
Ala side chains **A**					5 × 32		2 × 32
Gly side chains **G**		1 × 21			2 × 21		3 × 21
Tyr side chains **Y**					1 × 5375		
Leu side chains **L**					6 × 136		4 × 136
Total	81772	13983	8302	164623	26166	6252	53774

[a] Values according to ref. [2].

Binding Constants

Fluorescence titrations were performed as previously described and the resulting titration curves were analyzed using established procedures. Competitive titrations were either analyzed with a fitting function assuming 1:1 host-competitor complexes[3] or with a fitting function involving higher complexes with more than one host bound per peptide.[4] In the latter case, an identical binding affinity is assumed for all binding sites.

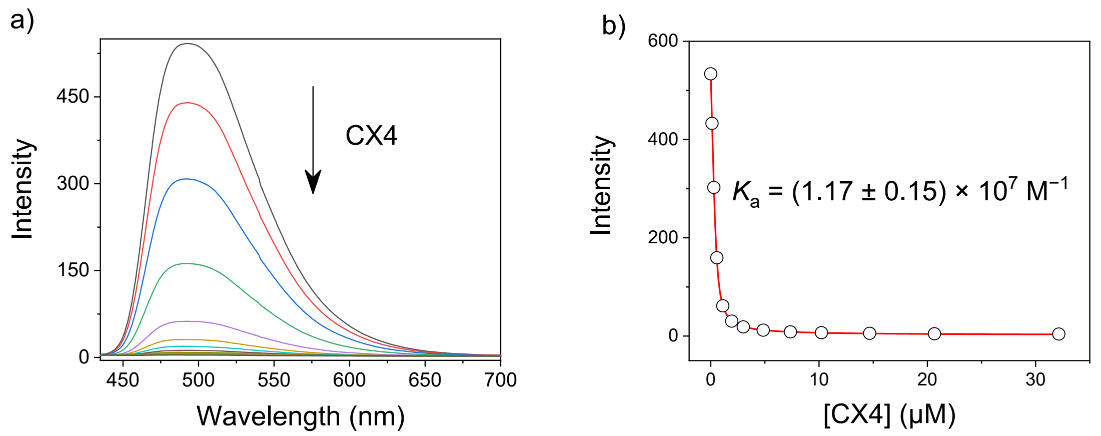

Figure S1. a) Fluorescence titration of 0.5 µM LCG (λ_{ex} = 369 nm) with varying concentrations of CX4 in 10 mM Hepes, pH 7.0, 25 °C. b) Respective titration curve (λ_{em} = 502 nm) with fitted line.

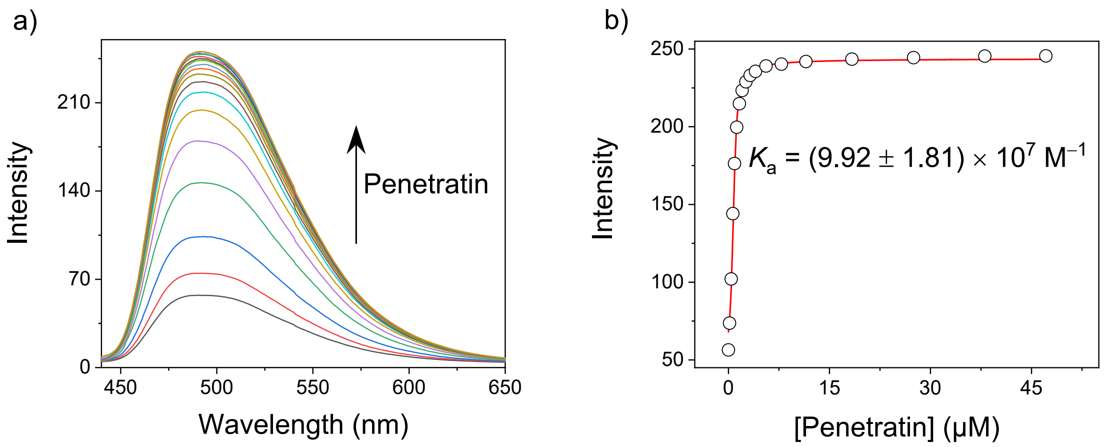

Figure S2. a) Competitive fluorescence titration with 0.5 µM LCG, 1 µM CX4 (λ_{ex} = 369 nm) and varying concentrations of penetratin in 10 mM Hepes, pH 7.0, 25 °C. b) Respective titration curve (λ_{em} = 502 nm) with fitted line.

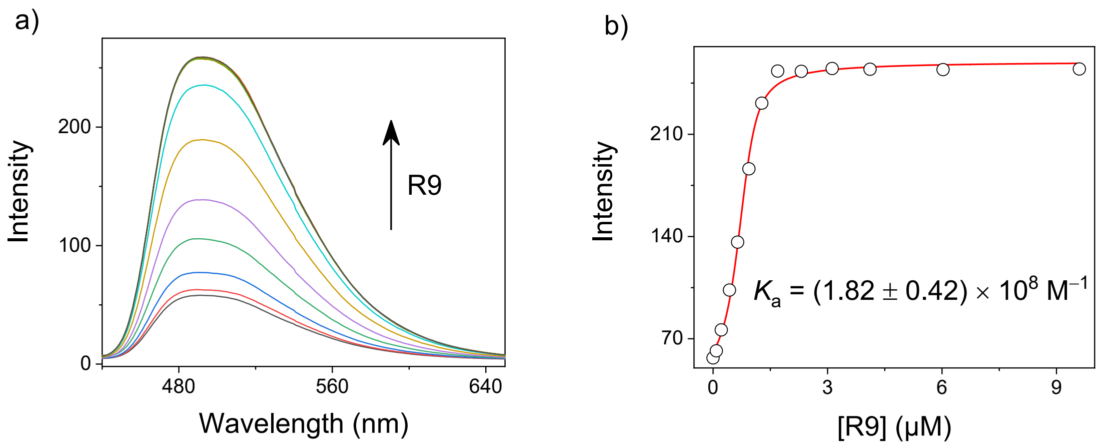

Figure S3. a) Competitive fluorescence titration with 0.5 μM LCG, 1 μM CX4 (λ_{ex} = 369 nm) and varying concentrations of R9 in 10 mM Hepes, pH 7.0, 25 °C. b) Respective titration curve (λ_{em} = 502 nm) with fitted line.

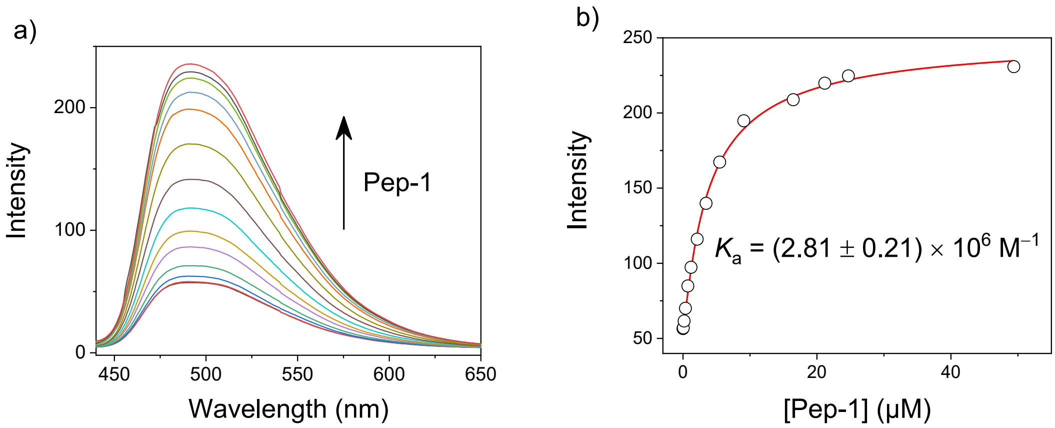

Figure S4. a) Competitive fluorescence titration with 0.5 μM LCG, 1 μM CX4 (λ_{ex} = 369 nm) and varying concentrations of Pep-1 in 10 mM Hepes, pH 7.0, 25 °C. b) Respective titration curve (λ_{em} = 502 nm) with fitted line.

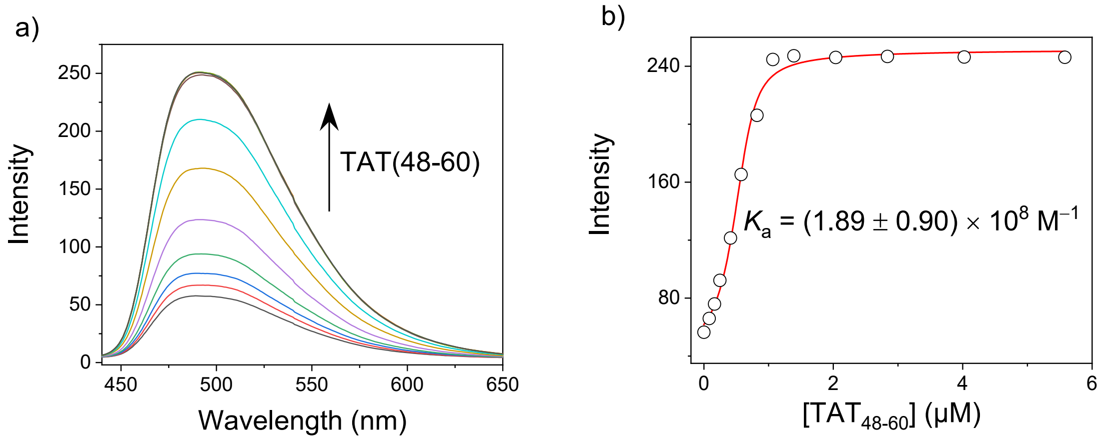

Figure S5. a) Competitive fluorescence titration with 0.5 μM LCG, 1 μM CX4 (λ_{ex} = 369 nm) and varying concentrations of TAT_{48-60} in 10 mM Hepes, pH 7.0, 25 °C. b) Respective titration curve (λ_{em} = 502 nm) with fitted line.

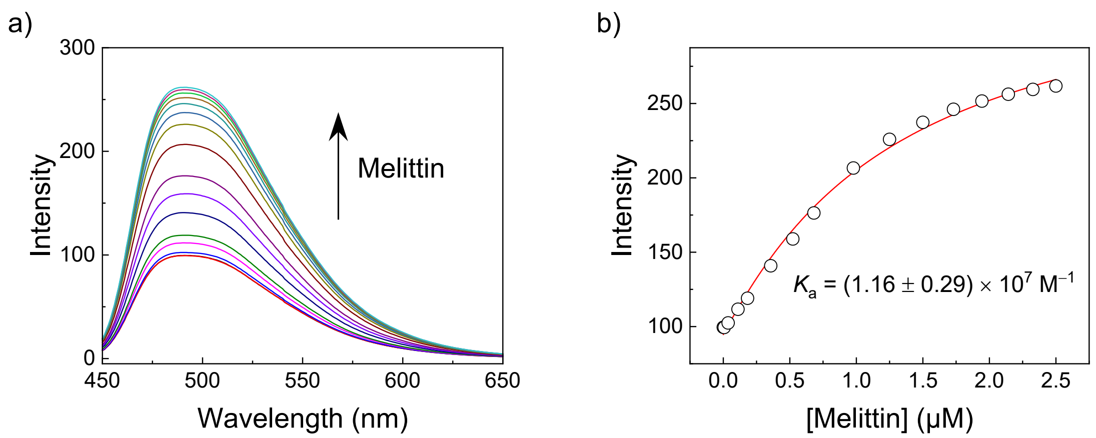

Figure S6. a) Competitive fluorescence titration with 1 μM LCG, 0.75 μM CX4 (λ_{ex} = 369 nm) and varying concentrations of melittin in 10 mM Hepes, pH 7.0, 25 °C. b) Respective titration curve (λ_{em} = 502 nm) with fitted line.

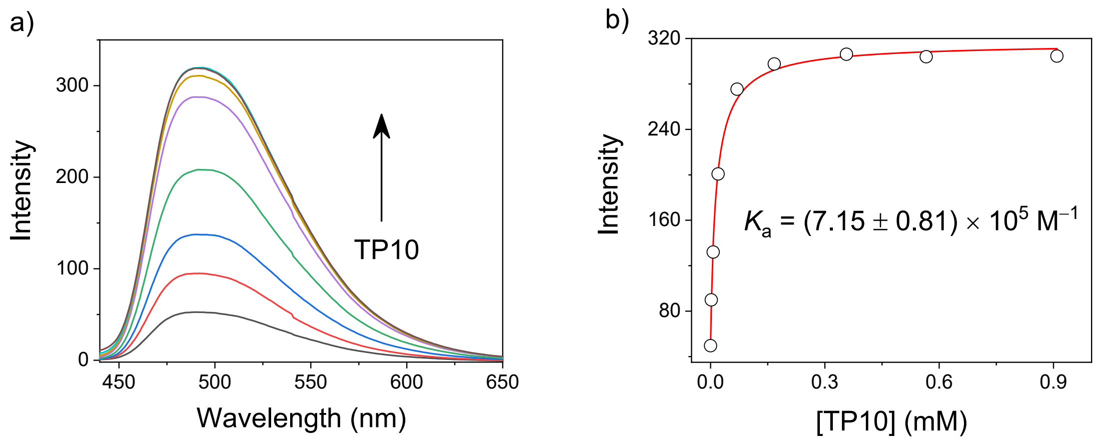

Figure S7. a) Competitive fluorescence titration with 0.5 μM LCG, 1.0 μM CX4 (λ_{ex} = 369 nm) and varying concentrations of TP10 in 10 mM Hepes, pH 7.0, 25 °C. b) Respective titration curve (λ_{em} = 490 nm) with fitted line.

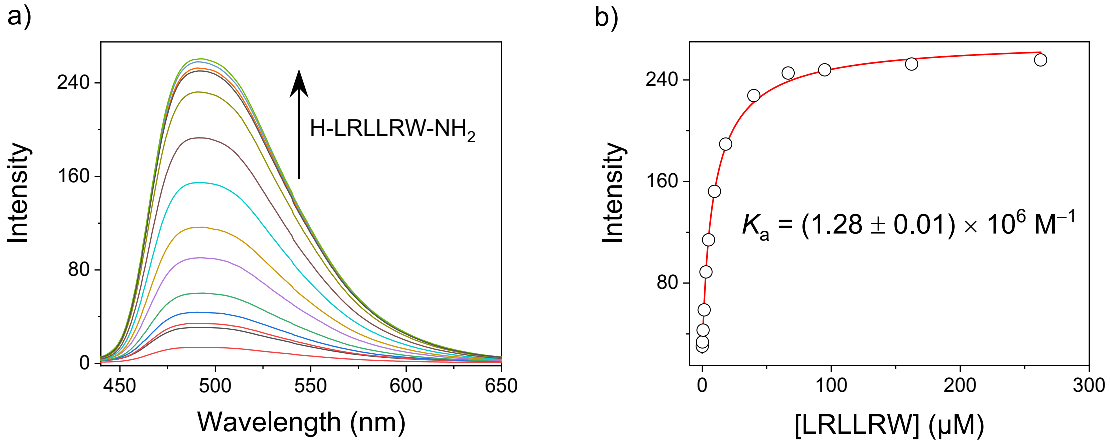

Figure S8. a) Competitive fluorescence titration with 0.5 μM LCG, 1 μM CX4 (λ_{ex} = 369 nm) and varying concentrations of LRLLRW in 10 mM Hepes, pH 7.0, 25 °C, b) Respective titration curve (λ_{em} = 502 nm) with fitted line.

Membrane Activity Measurements

Vesicle Preparation

CX4/LCG-LUVs

Vesicle preparation. 100 µL of a solution of 25 mg/ml of POPC in chloroform or 200 µL of a solution of 25 mg/ml of EYPC in chloroform was purged with nitrogen and dried overnight under high vacuum. The thin lipid film was rehydrated with 1 ml rehydration buffer (= inside buffer, see Table S2) by agitation at room temperature for 30 min and then subjected to 20 freeze-thaw cycles (freezing in liquid nitrogen and thawing at 40 °C). The resulting CX4/LCG-loaded liposomes were separated from unencapsulated CX4 and LCG by size exclusion chromatography using a 1.5 × 12 cm polypropylene column (Bio-Rad, Germany) with 1.0 g Sephadex G-50 (medium) as a stationary phase and the respective outside buffer for elution.

Table S2. Combinations of lipids and inside and outside buffer for CX4/LCG-LUVs.

No.	Lipid	Inside buffer = rehydration buffer	Outside buffer
(1)	POPC	10 mM Hepes, 700 µM CX4, 500 µM LCG, pH 7.0	10 mM Hepes, pH 7.0
(2)	POPC	10 mM Hepes, 700 µM CX4, 500 µM LCG, pH 7.5	10 mM Hepes, pH 7.5
(3)	EYPC	10 mM Hepes, 700 µM CX4, 500 µM LCG, pH 7.5	10 mM Hepes, pH 7.5
(4)	POPC	10 mM Hepes, 700 µM CX4, 500 µM LCG, 175 mM glucose, pH 7.5 [a]	10 mM Hepes buffer, 175 mM glucose, pH 7.5 [a]

[a] Glucose buffers (183 mOsm/L at 25 °C) were used within one week after preparation.

Vesicle quality control. To ensure the absence of the CX4/LCG reporter pair in the extravesicular phase, 20 µL of the collected liposome fraction was diluted to 2 ml with outside buffer and the fluorescence (λ_{exc} = 369 nm; λ_{em} = 502 nm) was recorded during the addition of 20 µL 2.5 mM spermine to afford 25 µM final spermine concentration. A constant fluorescence intensity during the addition of the strongly binding spermine verified the complete removal of unencapsulated material. The size distribution of the liposomes was confirmed by DLS to be unimodal and in the range of 120 to 150 nm diameter. The final lipid content in the liposome suspension after preparation and separation was determined by NMR as previously reported.[5]

Assay protocol. An appropriate volume of the liposome stock solutions was diluted to 2 mL with outside buffer in a 10×10 mm quartz glass cuvette to afford a phospholipid concentration of 25 µM. The cuvette was placed into a thermostatted fluorimeter sample holder at 25.0 °C and gently stirred at 300 rpm. Subsequently, the time-dependent change in fluorescence intensity (λ_{exc} = 369 nm; λ_{em} = 502 nm) was recorded during addition of 20 µL of peptide stock solutions of varying concentrations at 60 s. At 600 s, 20 µL of a solution containing 1.6% (wt/vol) TX-100 and 1.5 mM protamine was added for calibration to afford 0.016% (wt/vol) TX-100 and 15 µM protamine final concentrations.

Time-dependent fluorescence intensity traces, $I(t)$, were subsequently normalized to fractional intensities, $I_f(t)$, using equation (1). Therein, I_0 is the intensity just before addition of peptide and I_∞ is the constant intensity after addition of the calibration cocktail.

$$I_{\mathrm{f}}(t) = \frac{I(t) - I_0}{I_\infty - I_0} \qquad (1)$$

The concentration-dependent fractional intensities just before the addition of the calibration cocktail, I_f, were then normalized to fractional membrane activities, Y, using equation (2). Therein, $I_{f,0}$ is the fractional intensity just before addition of the calibration cocktail in absence of peptide and $I_{f,\infty}$ is the constant fractional intensity after addition of the calibration cocktail.

$$Y = \frac{I_{\mathrm{f}} - I_{\mathrm{f},0}}{I_{\mathrm{f},\infty} - I_{\mathrm{f},0}} \qquad (2)$$

The fractional membrane activities, Y, were subsequently plotted against the peptide concentration, c, and analysed using the Hill equation (3) to obtain the effective molar concentration, EC_{50}, and the Hill coefficient, n. Y_0 and Y_{max} are the fractional membrane activities in absence of peptide and at maximal peptide concentrations. For peptides with too little activity to determine Y_{max} experimentally, Y_{max} was fixed at 1.0 during fitting.

$$Y = Y_0 + \frac{Y_{\max} - Y_0}{1 + \left(\frac{c}{EC_{50}}\right)^n} \qquad (3)$$

CF-LUVs

Vesicle preparation. 100 µL of a solution of 25 mg/ml of POPC in chloroform or 200 µL of a solution of 25 mg/ml of EYPC in chloroform was purged with a stream of nitrogen and dried overnight under high vacuum. The thin lipid film was rehydrated with 1 ml of rehydration buffer (= inside buffer, see Table S3) by agitation at room temperature for 30 min and then subjected to 5 freeze-thaw cycles (freezing in liquid nitrogen and thawing at 40 °C). Subsequently, the liposome suspension was extruded ≥15 times through a polycarbonate membrane (pore size 100 nm). Extravesicular components were then removed by size exclusion chromatography using a 1.5 × 12 cm polypropylene column (Bio-Rad, Germany) with 1.0 g Sephadex G-50 (medium) as a stationary phase and the respective outside buffer for elution.

Table S3. Combinations of inside and outside buffer for CF-LUVs.

No.	Lipid	Inside buffer = rehydration buffer	Outside buffer
(1)	EYPC	10 mM Hepes, 50 mM CF, pH 7.5	10 mM Hepes, 97 mM NaCl, pH 7.5
(2)	POPC	10 mM Hepes, 50 mM CF, pH 7.5	10 mM Hepes, 97 mM NaCl, pH 7.5
(3)	POPC	10 mM Hepes, 50 mM CF, pH 7.5	10 mM Hepes, 175 mM glucose, pH 7.5 [a]

[a] Glucose buffers (183 mOsm/L at 25 °C) were used within one week after preparation.

Vesicle quality control. The size distribution of the liposomes was confirmed by DLS to be unimodal and average vesicles diameters were in the range from 115 to 130 nm. The final lipid content in the liposome suspension after preparation and separation was determined by NMR.[5] CF vesicles are comparably leaky and contain minor amounts of extravesicular CF after a few days. When the fluorescence change after vesicle lysis by addition of 20 µL 1.6% (wt/vol) TX-100 was less than a factor of four, the vesicle suspension was no longer used.

Assay protocol. An appropriate volume of the liposome stock solutions was diluted to 2 mL with outside buffer in a 10×10 mm quartz glass cuvette to afford a phospholipid concentration of 25 μM. The cuvette was placed into a thermostatted fluorimeter sample holder and gently stirred at 300 rpm at (25.0±0.1) °C. Subsequently, the time-dependent change in fluorescence intensity (CF: λ_{exc} = 492 nm, λ_{em} = 517 nm) was recorded during addition of 20 μL peptide stock solution of varying concentrations at 60 s and 20 μL of a calibration cocktail (1.6% (wt/vol) TX-100) at 600 s (final: 0.016% wt/vol) TX-100).

Time-dependent fluorescence intensity traces, $I(t)$, were subsequently normalized to fractional intensities, $I_f(t)$, using equation (1) and the concentration-dependent fractional intensities just before the addition of the calibration cocktail, I_f, were then normalized to fractional membrane activities, Y, using equation (2). The fractional membrane activities, Y, were subsequently plotted against the peptide concentration, c, and analysed using the Hill equation (3) to obtain the effective molar concentration, EC_{50}, and the Hill coefficient, n. For peptides with too low activity to determine Y_{max} experimentally, Y_{max} was fixed at 1.0 during fitting.

Osmotically Stressed POPC⊃CF-LUVs

Vesicles were prepared as described above and eluted with 10 mM Hepes, 97 mM NaCl, pH 7.5 in the size exclusion chromatography to store the vesicle solutions under osmotically balanced conditions. For the measurements, an appropriate volume of the liposome stock solutions was diluted to 2 mL with 10 mM Hepes, pH 7.5 without NaCl and the measurements were performed and analysed as described above. The final NaCl concentration was ca. 1 mM during measurements and thus negligible in all cases.

Dual-Channel Measurements

Vesicles were prepared as described above (see entry (4) in Table S2 and entry (3) in Table S3) and eluted with freshly prepared 10 mM Hepes, 175 mM glucose, pH 7.5 in the size exclusion chromatography. For the measurements, an appropriate volume of the liposome stock solutions was diluted to 2 mL in a 10×10 mm PS cuvette with 10 mM Hepes buffer and 175 mM glucose at pH 7.5 to afford 12.5 μM POPC⊃CX4/LCG-LUVs and 12.5 μM POPC⊃CF-LUVs (25 μM POPC total). The cuvette was placed into a thermostatted fluorimeter sample holder at 25.0 °C and gently stirred at 300 rpm. The fluorescence intensity was simultaneously recorded in the CF channel (λ_{exc} = 525 nm, λ_{em} = 545 nm) and in the LCG channel (λ_{exc} = 369 nm, λ_{em} = 475 nm) with an excitation slit width of 5 nm and an emission slit width of 5 nm during addition of 20 μL peptide stock solution of varying concentrations at 60 s and 20 μL of a calibration cocktail (1.6% (wt/vol) TX-100; 2 mM spermine) at 600 s (final: 0.016% wt/vol) TX-100).

Activity Screening

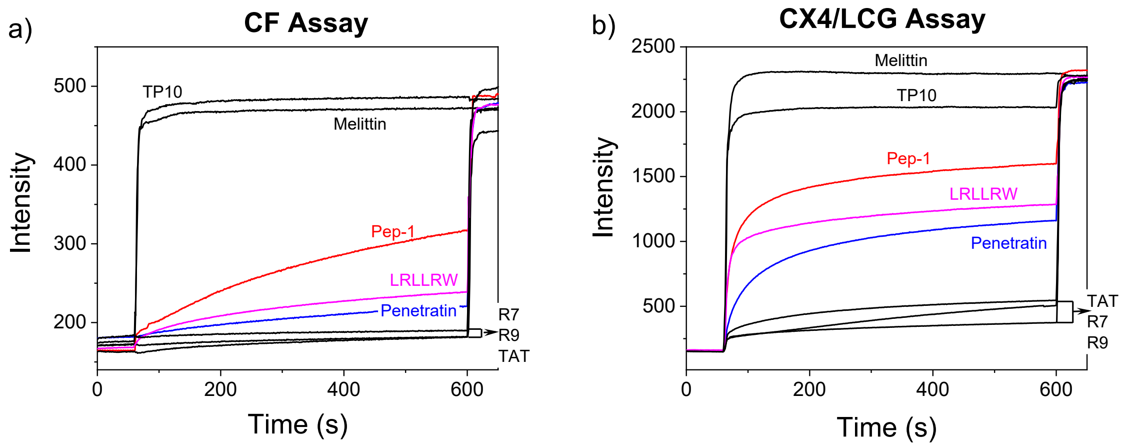

Figure S9. Original data of Fig. 1 in main manuscript: a) Time-dependent changes in fluorescence intensity of EYPC⊃CF-LUVs (λ_{exc} = 492 nm, λ_{em} =517 nm) in 10 mM Hepes, 97 mM NaCl, pH 7.5, 25 °C after addition 30 µM of different peptides (at 60 s), and TX-100 at 600 s. b) Time-dependent changes in fluorescence intensity of CX4/LCG-POPC-LUVs (λ_{ex} = 369 nm, λ_{em} = 502 nm) in 10 mM Hepes, pH 7.0 after addition of 30 µM of different peptides (at 60 s) and TX-100 and protamine for calibration (at 600 s).

Table S4. Membrane activity at 30 µM peptide concentrations with the CX4/LCG and CF assay.[a]

Peptide	$Y_{30\mu M}$	
	CF assay	CX4/LCG assay
melittin	1.00	1.00
TP10	1.00	0.88
Pep-1	0.46	0.67
Penetratin	0.14	0.49
R9	<0.10	0.11
TAT(48-60)	<0.10	0.19
R7	<0.10	0.17
LRLLRW	0.23	0.53

[a] CX4/LCG assay with 25 µM POPC⊃CX4/LCG-LUVs in 10 mM Hepes, pH 7.0; CF assay with 25 µM EYPC⊃CF-LUVs in 10 mM Hepes, 97 mM NaCl, pH 7.5. Peptide concentrations were 30 µM (n = 1).

Lipid Dependence

Table S5. Comparison of membrane activity with different lipids.[a]

Peptide	Assay	EC_{50} (μM)	
		EYPC	POPC
Pep-1	CF assay	35 ± 1	114 ± 5
	CX4/LCG assay	16.1 ± 1.4	32 ± 14
penetratin	CF assay	n.a. [b]	n.a. [b]
	CX4/LCG assay	14 ± 3	70 ± 13

[a] CF assay in 10 mM Hepes, 97 mM NaCl, pH 7.5. CX4/LCG assay in 10 mM Hepes, pH 7.5. Lipid concentrations were 25 μM. [b] The activity of penetratin in the CF assay was too low to determine an EC_{50} value. Errors correspond to the standard deviation obtained by nonlinear fitting (n = 1).

Pep-1: CF Assay and CX4/LCG Assay in POPC and EYPC

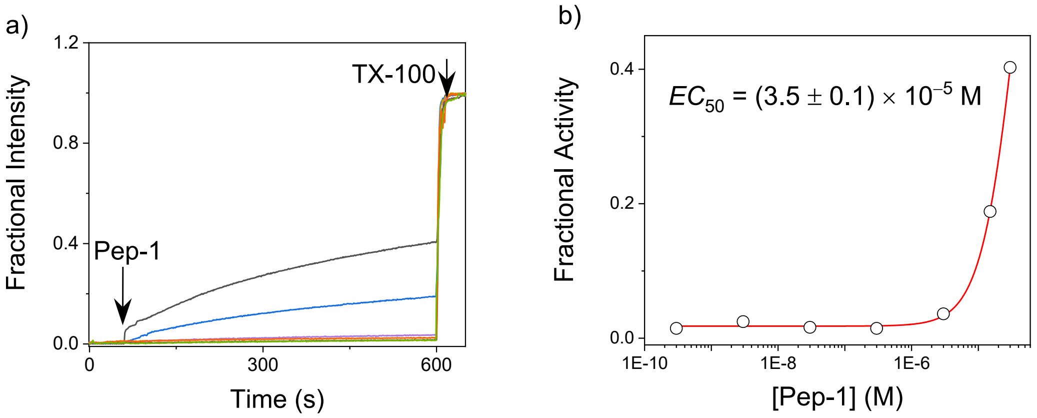

Figure S10. CF assay with Pep-1 in EYPC⊃CF-LUVs in 10 mM Hepes, 97 mM NaCl, pH 7.5, 25 °C. a) Time-dependent changes in fractional emission intensity of CF (λ_{ex} = 492 nm, λ_{em} = 517 nm) after addition of varying concentrations of Pep-1 (0.3 nM-30 μM) at 60 s and 20 μL 1.6% TX-100 after 600 s. b) Dependence of fractional activity at 590 s on Pep-1 concentration.

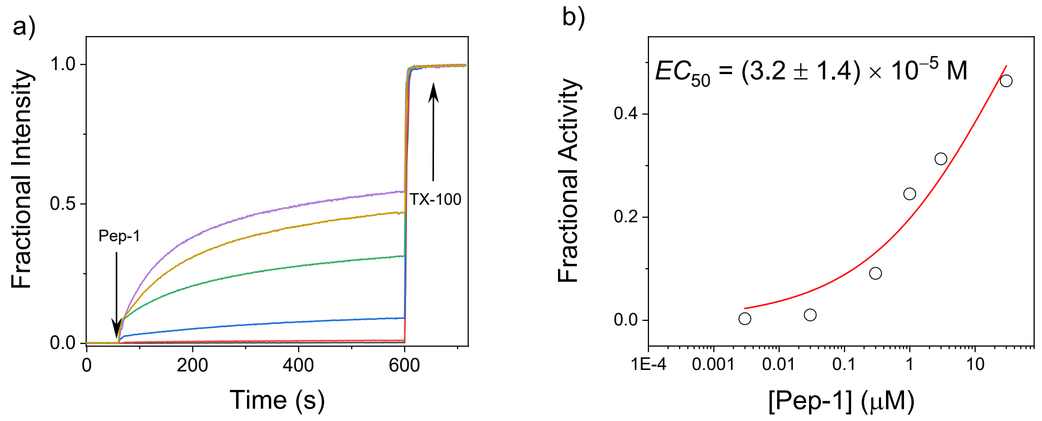

Figure S11. CX4/LCG assay with Pep-1 in POPC⊃CX4/LCG-LUVs in 10 mM Hepes, pH 7.5. a) Time-dependent changes in fractional emission intensity of LCG (λ_{ex} = 369 nm, λ_{em} = 502 nm) after addition of varying concentrations of Pep-1 (0.3 nM-30 μM) at 60 s and 20 μL of calibration cocktail (1.6% TX-100, 1.5 mM protamine) after 600 s. b) Dependence of fractional activity at 590 s on Pep-1 concentration.

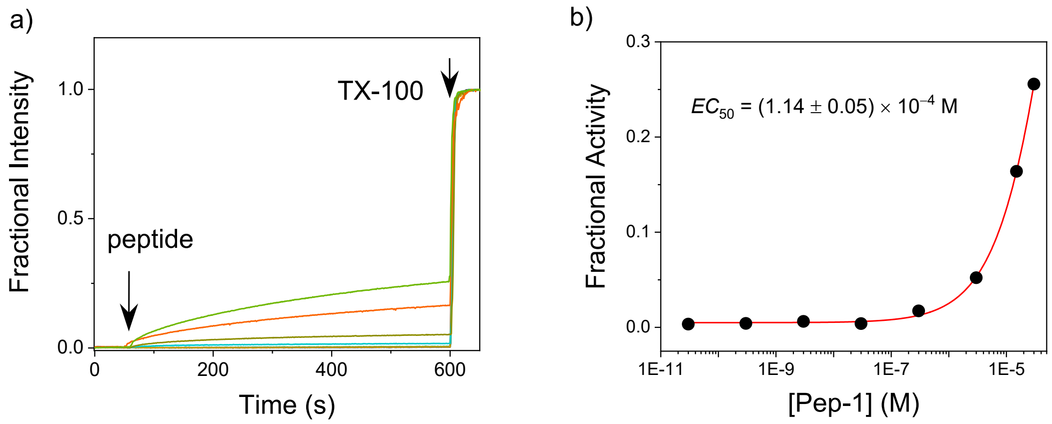

Figure S12. CF assay with Pep-1 and 25 μM POPC⊃CF-LUVs in 10 mM Hepes, 97 mM NaCl, pH 7.5, 25 °C. a) Time-dependent changes in fractional emission intensity of CF (λ_{ex} = 492 nm, λ_{em} = 517 nm) after addition of varying concentrations of Pep-1 at 60 s and 20 μL 1.6% TX-100 after 600 s. b) Dependence of fractional activity at 590 s on Pep-1 concentration.

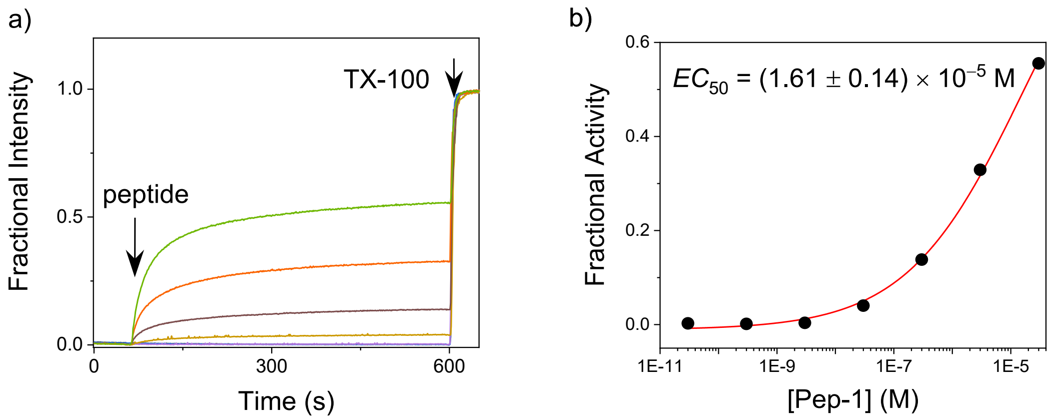

Figure S13. CX4/LCG assay with Pep-1 in EYPC⊃CX4/LCG-LUVs in 10 mM Hepes, pH 7.5, 25 °C. a) Time-dependent changes in fractional emission intensity of LCG (λ_{ex} = 369 nm, λ_{em} = 502 nm) after addition of varying concentrations of Pep-1 (3 pM to 30 μM) at 60 s and 20 μL of calibration cocktail (1.6% TX-100, 1.5 mM protamine) after 600 s. b) Dependence of fractional activity at 590 s on Pep-1 concentration.

Penetratin: CX4/LCG Assay in POPC and EYPC

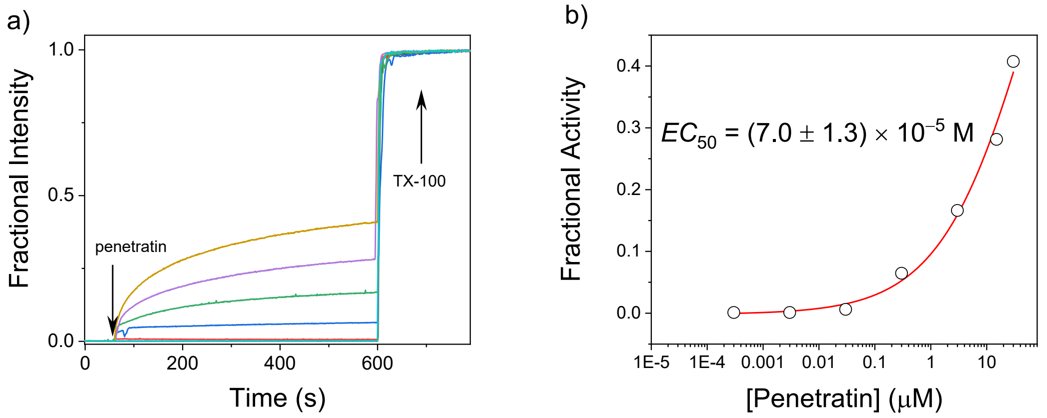

Figure S14. CX4/LCG assay with penetratin in POPC⊃CX4/LCG-LUVs in 10 mM Hepes, pH 7.5. a) Time-dependent changes in fractional emission intensity of LCG (λ_{ex} = 369 nm, λ_{em} = 502 nm) after addition of varying concentrations of penetratin at 60 s and 20 μL calibration cocktail (1.6% TX-100, 1.5 mM protamine) after 600 s. b) Dependence of fractional activity at 590 s on penetratin concentration.

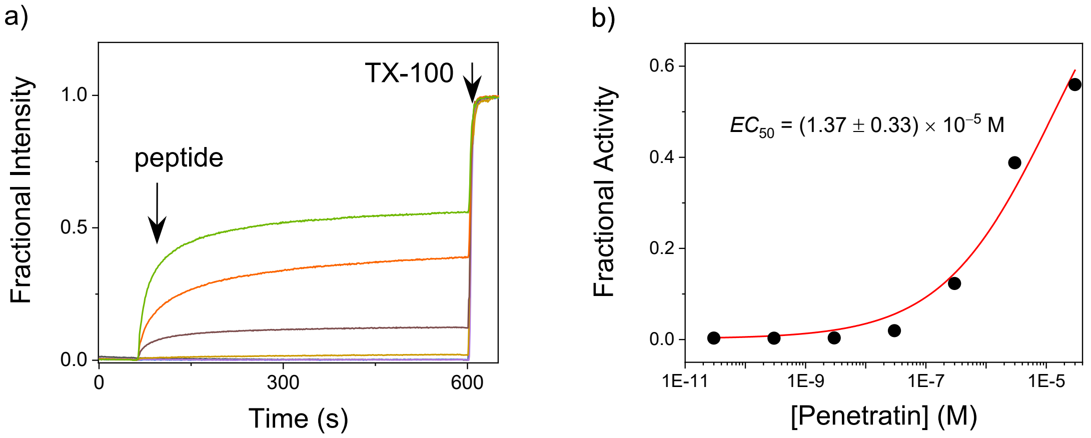

Figure S15. CX4/LCG assay with penetratin in EYPC⊃CX4/LCG-LUVs in 10 mM Hepes, pH 7.5. a) Time-dependent changes in fractional emission intensity of LCG (λ_{ex} = 369 nm, λ_{em} = 502 nm) after addition of varying concentrations of penetratin at 60 s and 20 μL calibration cocktail (1.6% TX-100, 1.5 mM protamine) after 600 s. b) Dependence of fractional activity at 590 s on penetratin concentration.

Influence of Osmotic Stress in the CF Assay

Chloride Quenching of LCG

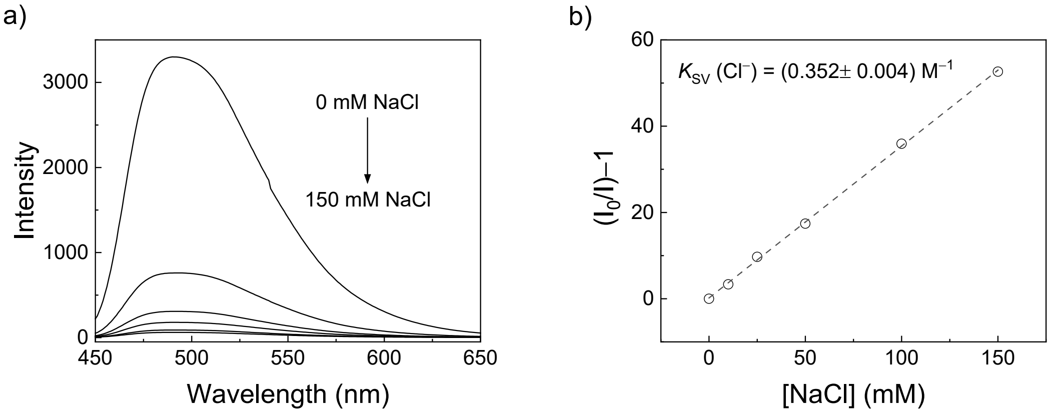

Figure S16. a) Fluorescence spectra (λ_{ex} = 369 nm) of LCG with different concentrations of Cl^- in 10 mM NaH_2PO_4, pH 7.2. b) Respective Stern-Volmer plot.

POPC⊃CF-LUVs with and without extravesicular NaCl

Table S6. Dependence of membrane activity on osmotic stress.[a]

Peptide	EC_{50} (M)	
	isosmotic	hypoosmotic
TP10	$(2.3 \pm 0.4) \times 10^{-6}$	$(6.3 \pm 0.7) \times 10^{-9}$
Pep-1	$(1.9 \pm 0.7) \times 10^{-4}$	$(1.9 \pm 0.5) \times 10^{-6}$
Penetratin	n.a. [b]	$(2.6 \pm 0.4) \times 10^{-8}$

[a] With the CF assay using 25 µM POPC⊃CF-LUVs diluted into 10 mM Hepes, 97 mM NaCl, pH 7.5 for isosmotic conditions and 10 mM Hepes, pH 7.5 for hypoosmotic conditions. [b] The activity under isosmotic conditions was too low to determine an EC_{50} value. Errors correspond to the standard deviation obtained by nonlinear fitting (n = 1).

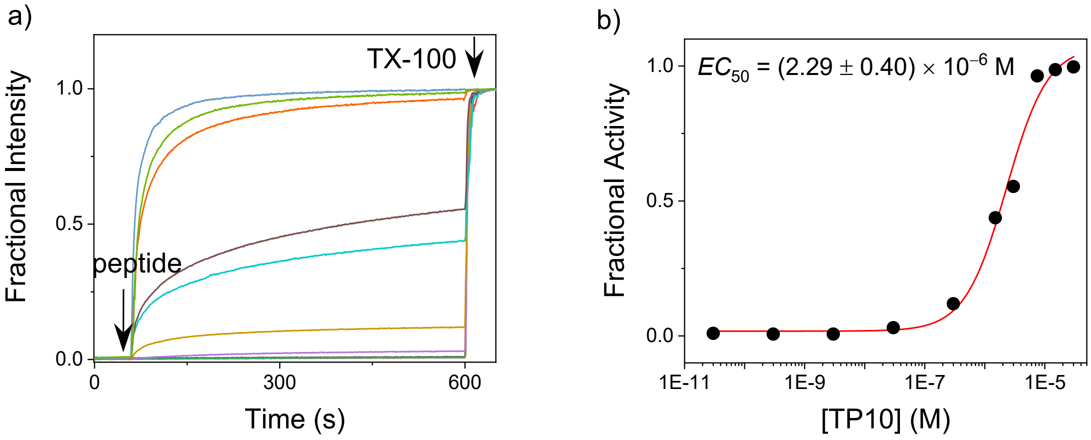

Figure S17. CF assay under isosmotic conditions with TP10 in POPC⊃CF-LUVs in 10 mM Hepes, 97 mM NaCl, pH 7.5, 25 °C. a) Time-dependent changes in fractional emission intensity of CF (λ_{ex} = 492 nm, λ_{em} = 517 nm) after addition of varying concentrations of TP10 at 60 s and 20 µL of 1.6% (wt/vol) TX-100 after 600 s. b) Dependence of fractional activity at 590 s on TP10 concentration.

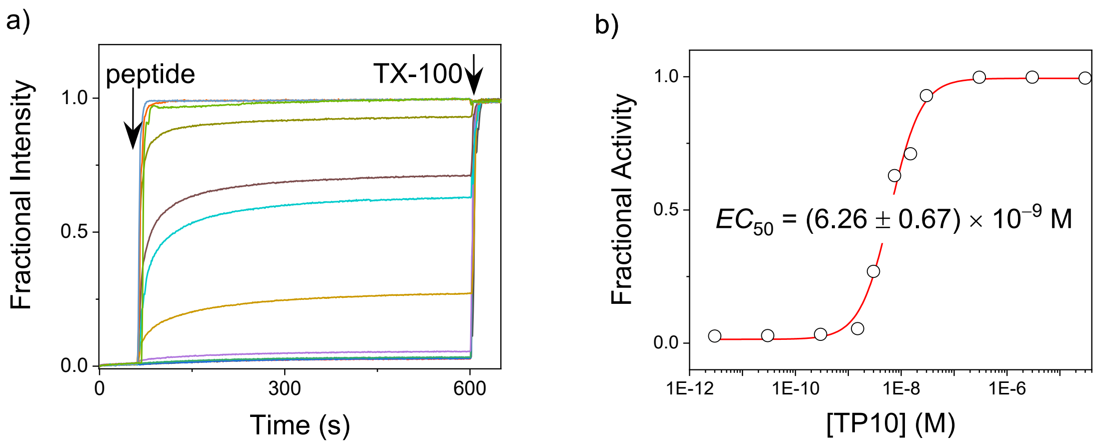

Figure S18. CF assay under hypoosmotic conditions with TP10 in POPC⊃CF-LUVs in 10 mM Hepes, pH 7.5, 25 °C. a) Time-dependent changes in fractional emission intensity of CF (λ_{ex} = 492 nm, λ_{em} = 517 nm) after addition of varying concentrations of TP10 at 60 s and 20 µL of 1.6% (wt/vol) TX-100 after 600 s. b) Dependence of fractional activity at 590 s on TP10 concentration.

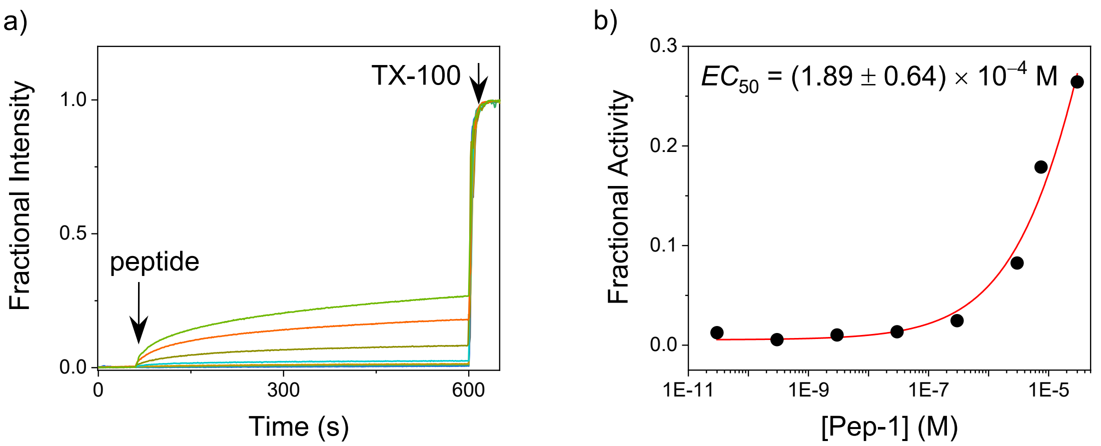

Figure S19. CF assay under isosmotic conditions with Pep-1 in POPC⊃CF-LUVs in 10 mM Hepes, 97 mM NaCl, pH 7.5, 25 °C. a) Time-dependent changes in fractional emission intensity of CF (λ_{ex} = 492 nm, λ_{em} = 517 nm) after addition of varying concentrations of Pep-1 at 60 s and 20 µL of 1.6% (wt/vol) TX-100 after 600 s. b) Dependence of fractional activity at 590 s on Pep-1 concentration.

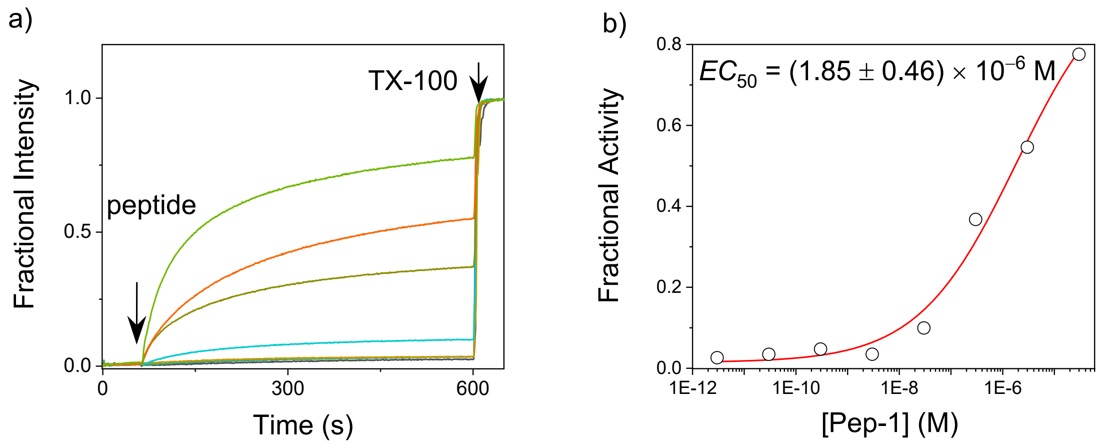

Figure S20. CF assay under hypoosmotic conditions with Pep-1 in POPC⊃CF-LUVs in 10 mM Hepes, pH 7.5, 25 °C. a) Time-dependent changes in fractional emission intensity of CF (λ_{ex} = 492 nm, λ_{em} = 517 nm) after addition of varying concentrations of Pep-1 at 60 s and 20 µL of 1.6% (wt/vol) TX-100 after 600 s. b) Dependence of fractional activity at 590 s on Pep-1 concentration.

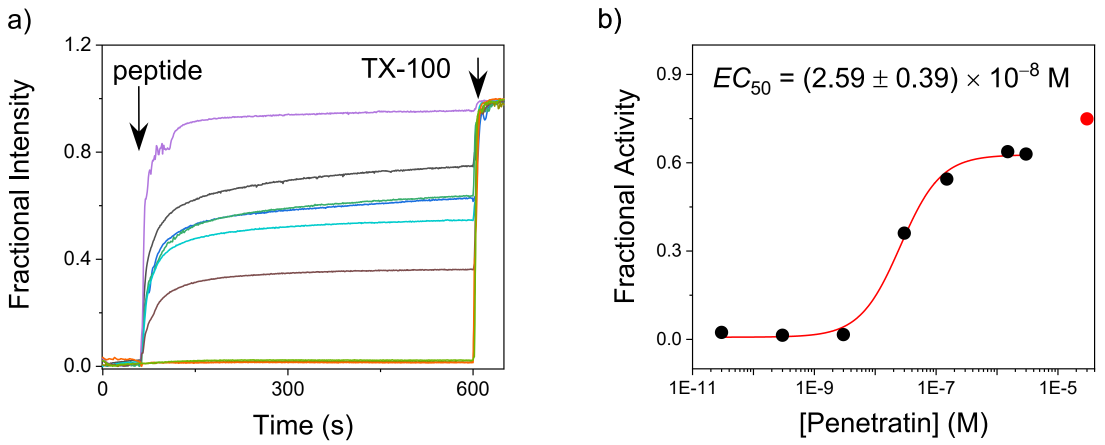

Figure S21. CF assay under hypoosmotic conditions with penetratin in POPC⊃CF-LUVs in 10 mM Hepes, pH 7.5, 25 °C. a) Time-dependent changes in fractional emission intensity of CF (λ_{exc} = 492 nm, λ_{em} = 517 nm) after addition of varying concentrations of penetratin at 60 s and 20 µL of 1.6% TX-100 (wt/v) after 600 s. b) Dependence of fractional activity at 590 s with varying penetratin concentrations. The data point marked in red was not included in the fitting.

Dual-Channel Assay Measurements

Influence of Glucose on CX4/LCG

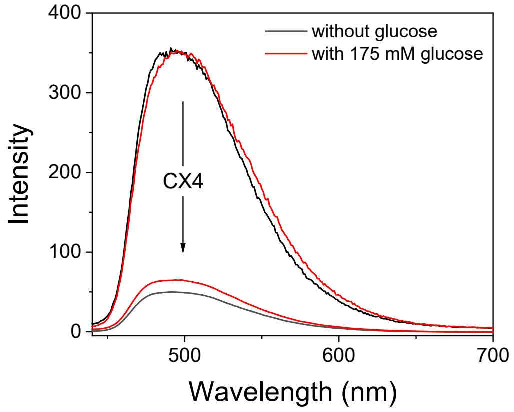

Figure S22. Influence of 175 mM glucose on the fluorescence of LCG and the CX4/LCG complex.

Assay Development

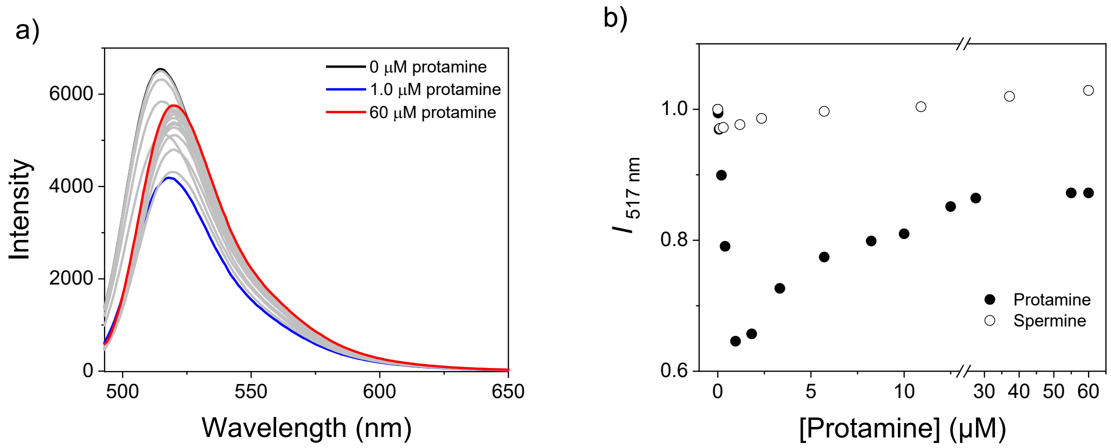

Figure S23. a) Fluorescence emission spectra of 1 µM CF (λ_{ex} = 492 nm) with varying concentrations of protamine in 10 mM Hepes, pH 7.5, 25 °C. b) Concentration dependence of protamine and spermine.

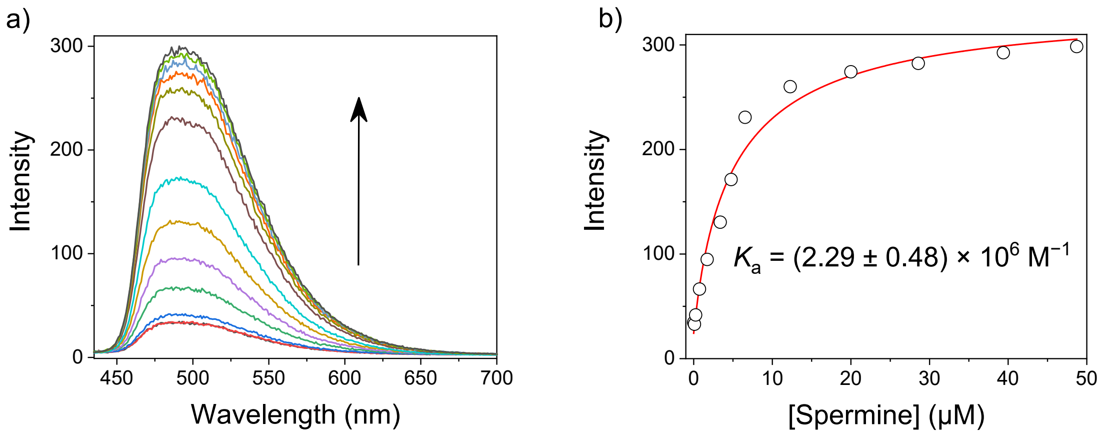

Figure S24. a) Competitive fluorescence titration with 0.5 µM LCG, 1 µM CX4 (λ_{ex} = 369 nm) and varying concentrations of spermine in 10 mM Hepes, pH 7.5, 25 °C. b) Respective fitting titration curve (λ_{em} = 502 nm).

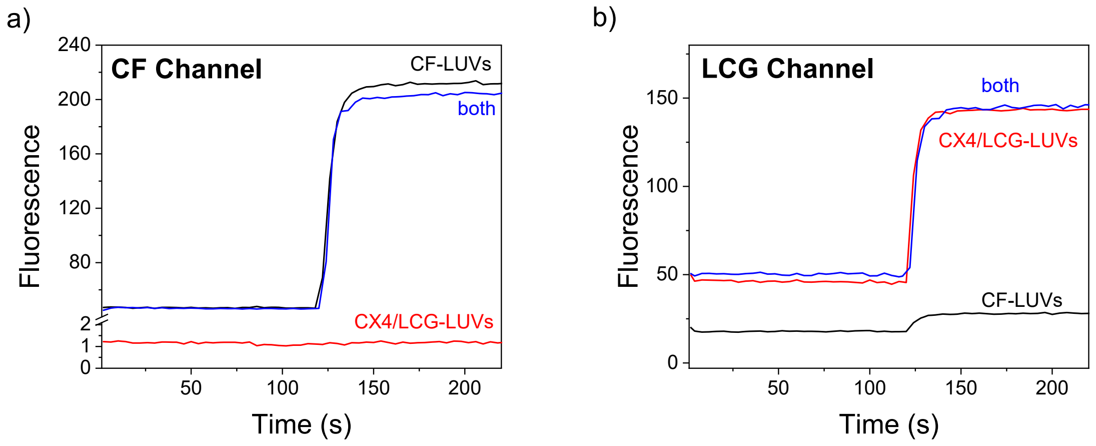

Figure S25. Vesicle lysis monitored a) in the CF channel (λ_{exc} = 525 nm, λ_{em} = 545 nm) and b) in the LCG channel (λ_{exc} = 369 nm, λ_{em} = 475 nm) with CF-LUVs (black line), CX4/LCG-LUVs (red line), or both (blue line). At 120 s, 20 µL of a solution containing 1.6% (wt/vol) TX-100 and 2 mM spermine was added for calibration (final concentrations: 0.016% (wt/vol) TX-100 and 20 µM spermine).

CX4/LCG Assay with Glucose Buffer

Table S7. Membrane activity of peptides with the CX4/LCG assay in glucose-containing buffer.[a]

Peptide	EC_{50} (µM)
Melittin	0.95 ± 0.02
TP10	1.7 ± 0.6
Pep-1	4.5 ± 0.7
Penetratin	140 ± 40
LRLLRW	14 ± 2

[a] 25 µM POPC⊃CX4/LCG-LUVs. Inside: 10 mM Hepes, 700 µM CX4, 500 µM LCG, 175 mM glucose, pH 7.5. Outside: 10 mM Hepes, 175 mM glucose, pH 7.5. Errors correspond to the standard deviation obtained by nonlinear fitting (n = 1).

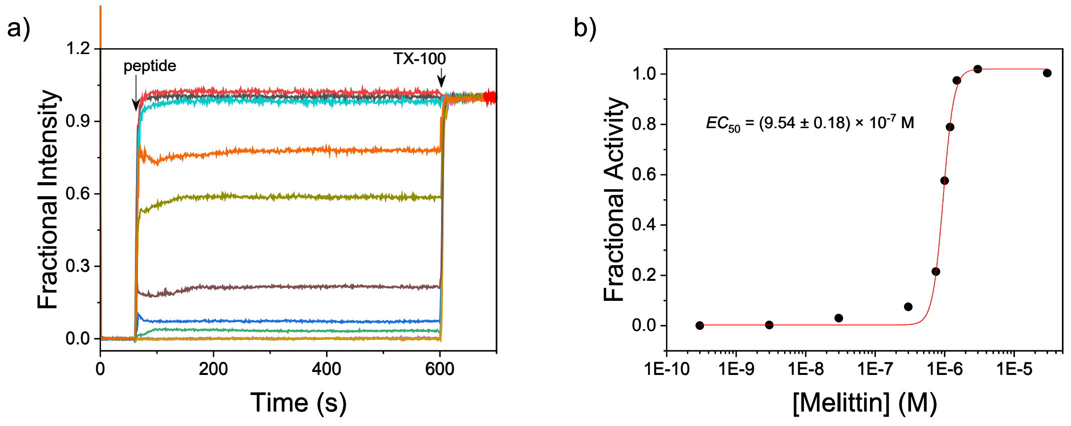

Figure S26. CX4/LCG assay with melittin with POPC⊃CX4/LCG-LUVs in 10 mM Hepes, 175 mM glucose, pH 7.5. a) Change in fractional emission intensity of LCG (λ_{ex} = 369 nm, λ_{em} = 502 nm) after addition of varying concentrations of melittin at 60 s and 20 µL calibration cocktail (1.6% TX-100 and 1.5 mM protamine) at 600 s. b) Fractional activity at 590 s with varying concentrations of melittin.

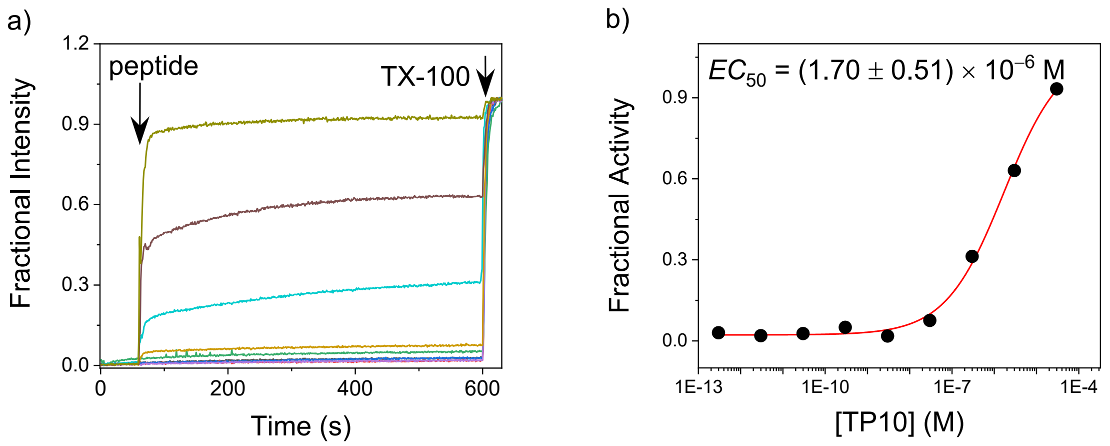

Figure S27. CX4/LCG assay with TP10 with POPC⊃CX4/LCG-LUVs in 10 mM Hepes, 175 mM glucose, pH 7.5. a) Change in fractional emission intensity of LCG (λ_{ex} = 369 nm, λ_{em} = 502 nm) after addition of varying concentrations of TP10 at 60 s and 20 µL calibration cocktail (1.6% TX-100 and 1.5 mM protamine) at 600 s. b) Fractional activity at 590 s with varying concentrations of TP10.

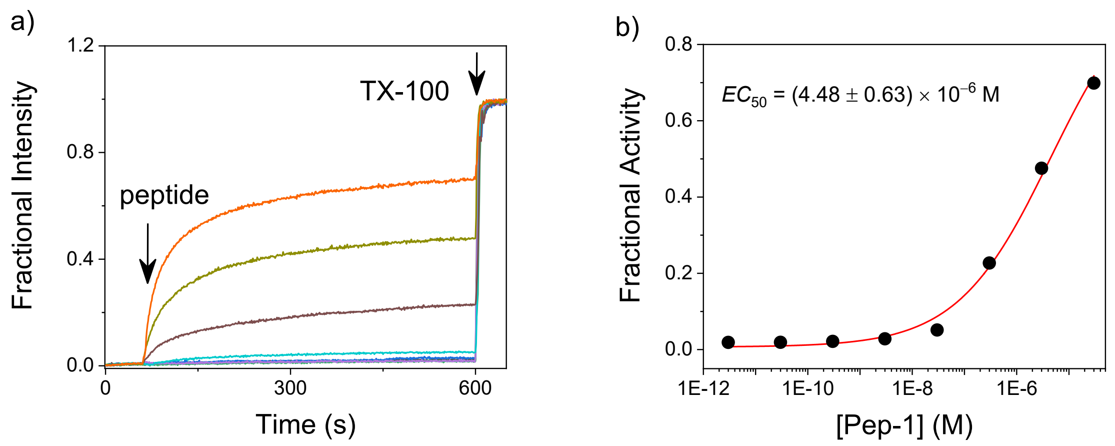

Figure S28. CX4/LCG assay with Pep-1 with POPC⊃CX4/LCG-LUVs in 10 mM Hepes, 175 mM glucose, pH 7.5. a) Change in fractional emission intensity of LCG (λ_{ex} = 369 nm, λ_{em} = 502 nm) after addition of varying concentrations of Pep-1 at 60 s and 20 µL calibration cocktail (1.6% TX-100 and 1.5 mM protamine) at 600 s. b) Fractional activity at 590 s with varying concentrations of Pep-1.

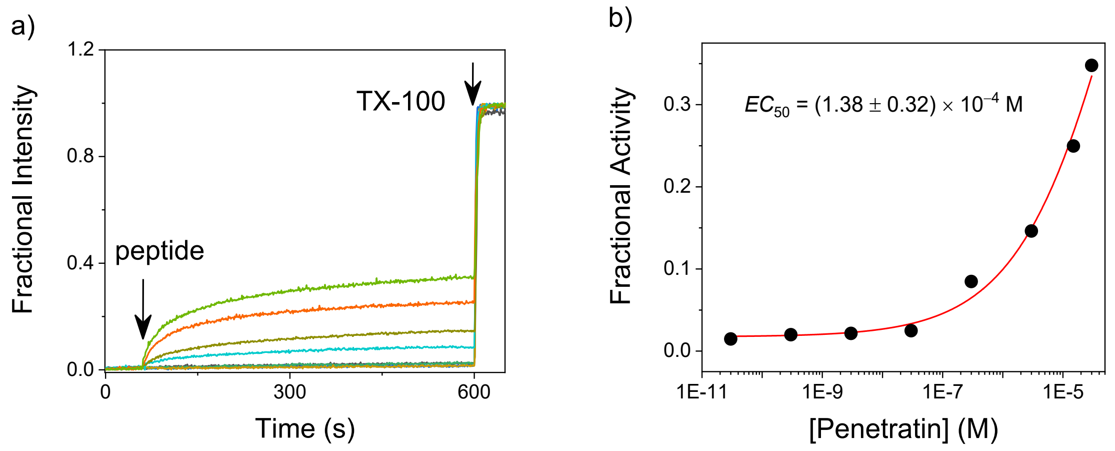

Figure S29. CX4/LCG assay with penetratin with POPC⊃CX4/LCG-LUVs in 10 mM Hepes, 175 mM glucose, pH 7.5. a) Change in fractional emission intensity of LCG (λ_{ex} = 369 nm, λ_{em} = 502 nm) after addition of varying concentrations of penetratin at 60 s and 20 µL calibration cocktail (1.6% TX-100 and 1.5 mM protamine) at 600 s. b) Fractional activity at 590 s with varying concentrations of penetratin.

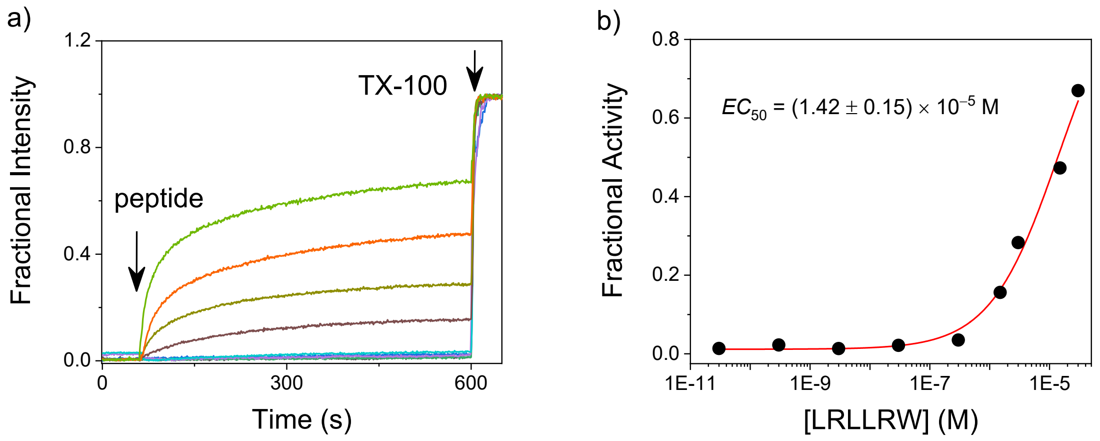

Figure S30. CX4/LCG assay with LRLLRW with POPC⊃CX4/LCG-LUVs in 10 mM Hepes, 175 mM glucose, pH 7.5. a) Change in fractional emission intensity of LCG (λ_{ex} = 369 nm, λ_{em} = 502 nm) after addition of varying concentrations of LRLLRW at 60 s and 20 μL calibration cocktail (1.6% TX-100 and 1.5 mM protamine) at 600 s. b) Fractional activity at 590 s with varying concentrations of LRLLRW.

CF Assay with Glucose Buffer

Table S8. Membrane activity of peptides with the CF assay in glucose-containing buffer.[a]

Peptide	EC_{50} (μM)
Melittin	0.017 ± 0.001
TP10	0.12 ± 0.02
Pep-1	540 ± 90
Penetratin	n.d.
LRLLRW	101 ± 9

[a] 25 μM POPC⊃CF-LUVs. Inside: 10 mM Hepes, 50 mM CF, pH 7.5. Outside: 10 mM Hepes, 175 mM glucose, pH 7.5. Errors correspond to the standard deviation obtained by nonlinear fitting (n = 1).

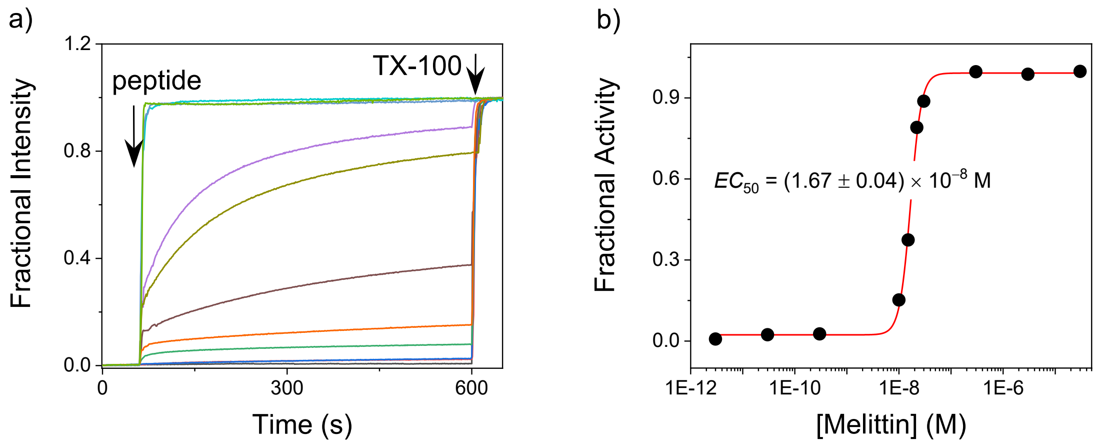

Figure S31. CF assay with melittin in CF⊃POPC-LUVs in 10 mM Hepes, 175 mM glucose, pH 7.5. a) Change in fractional emission intensity of CF (λ_{ex} = 492 nm, λ_{em} = 517 nm) after addition of varying concentrations of melittin at 60 s and 20 μL of 1.6% TX-100 at 600 s. b) Fractional activity at 590 s with varying concentrations of melittin.

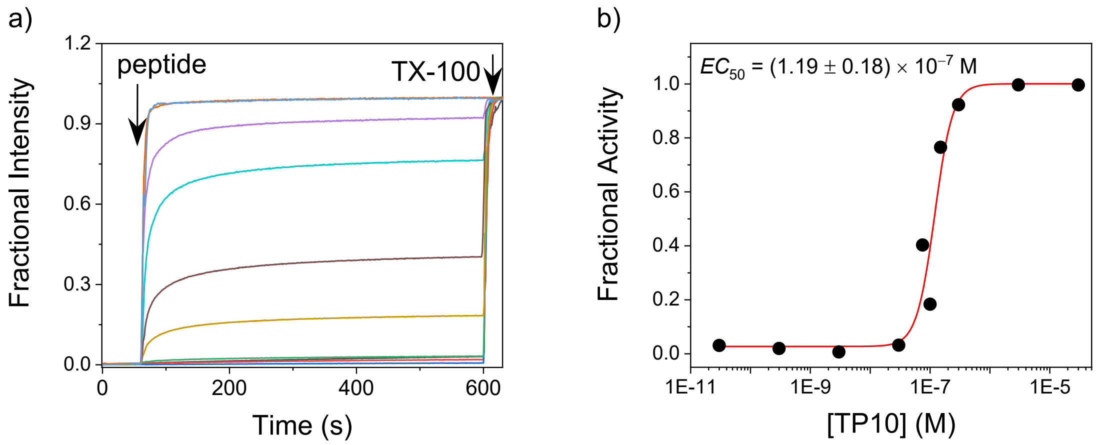

Figure S32. CF assay with TP10 in CF⊃POPC-LUVs in 10 mM Hepes, 175 mM glucose, pH 7.5. a) Change in fractional emission intensity of CF (λ_{ex} = 492 nm, λ_{em} = 517 nm) after addition of varying concentrations of TP10 at 60 s and 20 μL of 1.6% TX-100 at 600 s. b) Fractional activity at 590 s with varying concentrations of TP10.

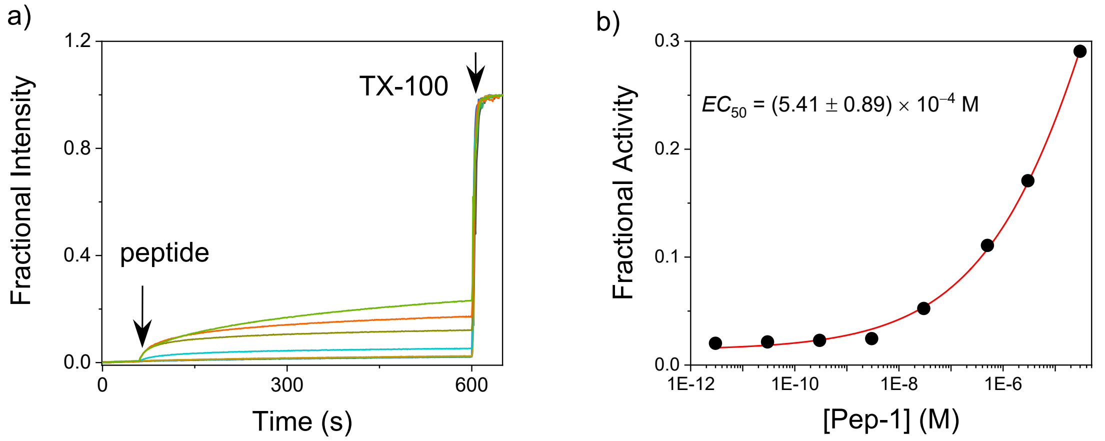

Figure S33. CF assay with Pep-1 in CF⊃POPC-LUVs in 10 mM Hepes, 175 mM glucose, pH 7.5. a) Change in fractional emission intensity of CF (λ_{ex} = 492 nm, λ_{em} = 517 nm) after addition of varying concentrations of Pep-1 at 60 s and 20 µL of 1.6% TX-100 at 600 s. b) Fractional activity at 590 s with varying concentrations of Pep-1.

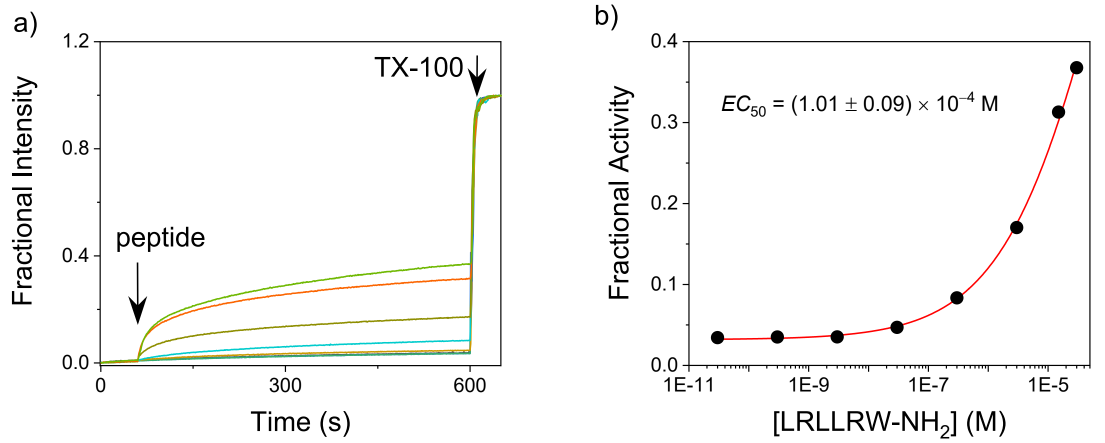

Figure S34. CF assay with LRLLRW in CF⊃POPC-LUVs in 10 mM Hepes, 175 mM glucose, pH 7.5. a) Change in fractional emission intensity of CF (λ_{ex} = 492 nm, λ_{em} = 517 nm) after addition of varying concentrations of LRLLRW at 60 s and 20 µL of 1.6% TX-100 at 600 s. b) Fractional activity at 590 s with varying concentrations of LRLLRW.

Dual-Channel Measurements

Table S9. Membrane activity of peptides determined by the dual-channel assay.[a]

Peptide	EC_{50} (µM)	
	CX4/LCG channel	CF assay
Melittin	0.066 ± 0.010	0.0034 ± 0.0002
TP10	0.78 ± 0.09	0.053 ± 0.007
Pep-1	22 ± 16	>1000
Penetratin	28 ± 2	>1000
LRLLRW	9.0 ± 0.9	49 ± 6

[a] Measured with 12.5 µM POPC⊃CX4/LCG-LUVs and 12.5 µM POPC⊃CF-LUVs in 10 mM Hepes, 175 mM glucose, pH 7.5. Errors correspond to the standard deviation obtained by nonlinear fitting (n = 1).

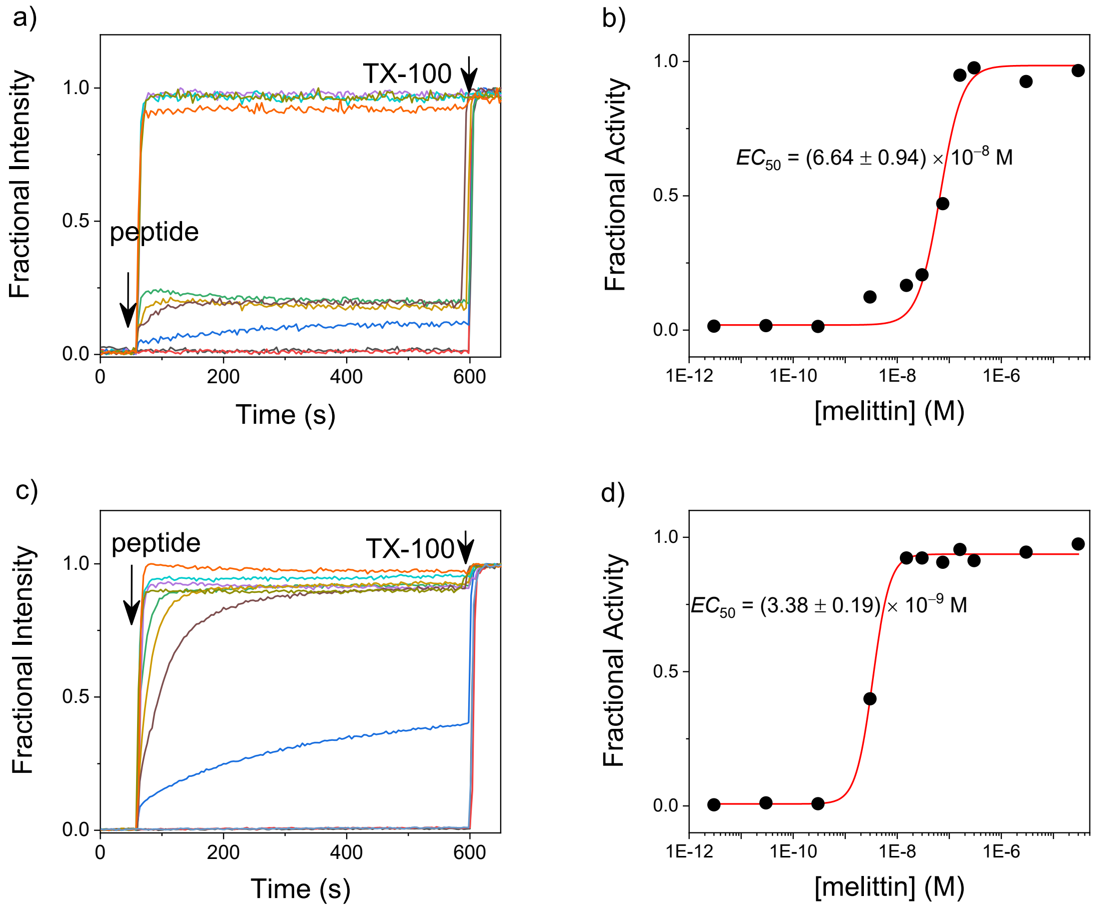

Figure S35. Dual-channel assay with melittin with POPC⊃CX4/LCG- and POPC⊃CF-LUVs (12.5 µM each) in 10 mM Hepes, 175 mM glucose, pH 7.5. a) Change in fractional emission intensity of the CX4/LCG channel (λ_{ex} = 369 nm; λ_{em} = 475 nm) and b) respective plot of fractional activity at 590 s with varying concentrations of melittin. c) Change in fractional emission intensity of the CF channel (λ_{ex} = 525 nm: λ_{em} = 545 nm) and d) respective plot of fractional activity at 590 s with varying concentrations of melittin. In each experiment, varying concentrations of melittin were added after 60 s and 20 µL of 1.6% TX-100 with 2 mM spermine for calibration after 600 s.

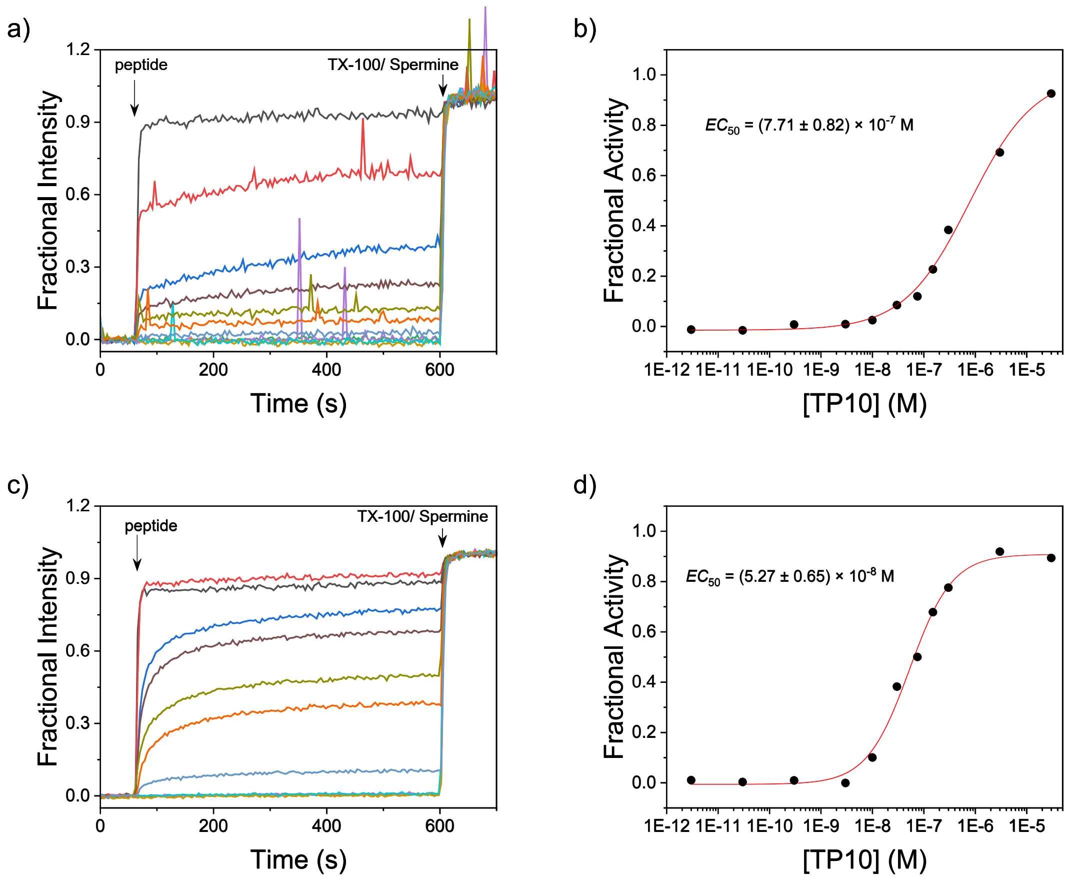

Figure S36. Dual-channel assay with TP10 with POPC⊃CX4/LCG- and POPC⊃CF-LUVs (12.5 µM each) in 10 mM Hepes, 175 mM glucose, pH 7.5. a) Change in fractional emission intensity of the CX4/LCG channel (λ_{ex} = 369 nm; λ_{em} = 475 nm) and b) respective plot of fractional activity at 590 s with varying concentrations of TP10. c) Change in fractional emission intensity of the CF channel (λ_{ex} = 525 nm: λ_{em} = 545 nm) and d) respective plot of fractional activity at 590 s with varying concentrations of TP10. In each experiment, varying concentrations of TP10 were added after 60 s and 20 µL of 1.6% TX-100 with 2 mM spermine for calibration after 600 s.

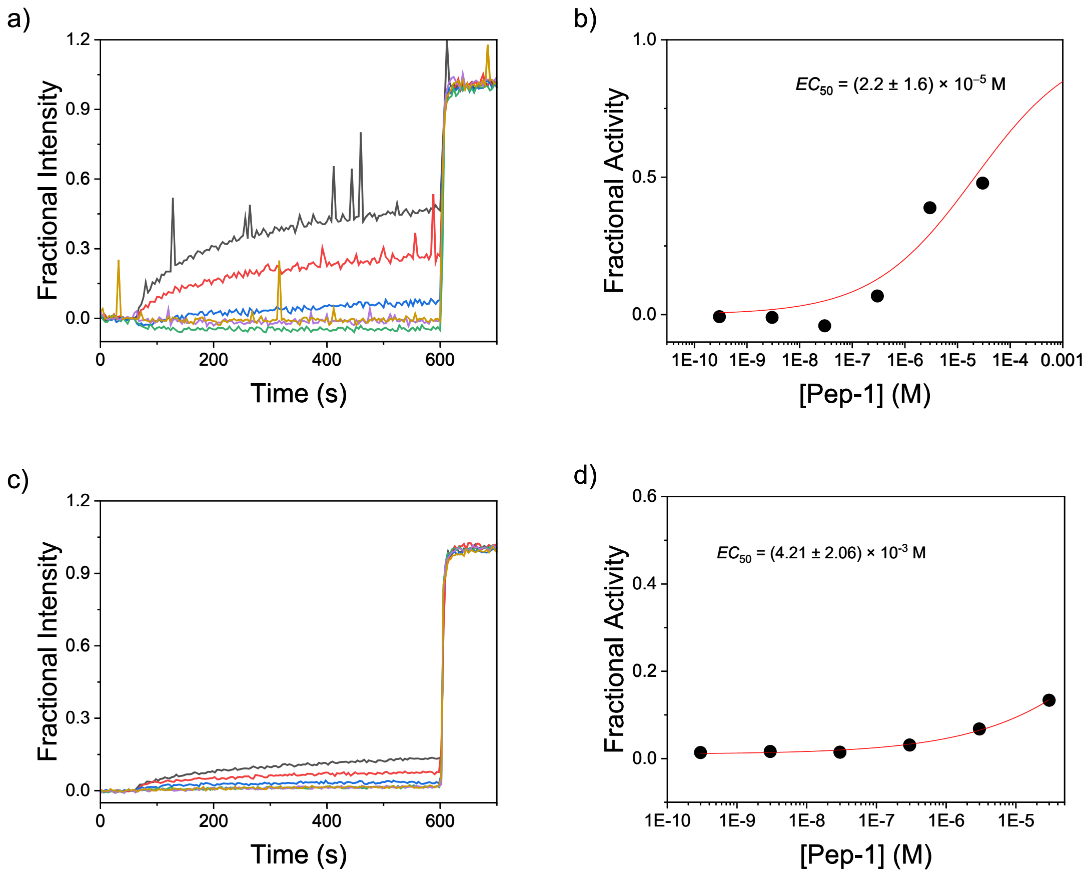

Figure S37. Dual-channel assay with Pep-1 with POPC⊃CX4/LCG- and POPC⊃CF-LUVs (12.5 µM each) in 10 mM Hepes, 175 mM glucose, pH 7.5. a) Change in fractional emission intensity of the CX4/LCG channel (λ_{ex} = 369 nm; λ_{em} = 475 nm) and b) respective plot of fractional activity at 590 s with varying concentrations of Pep-1. c) Change in fractional emission intensity of the CF channel (λ_{ex} = 525 nm: λ_{em} = 545 nm) and d) respective plot of fractional activity at 590 s with varying concentrations of Pep-1. In each experiment, varying concentrations of Pep-1 were added after 60 s and 20 µL of 1.6% TX-100 with 2 mM spermine for calibration after 600 s.

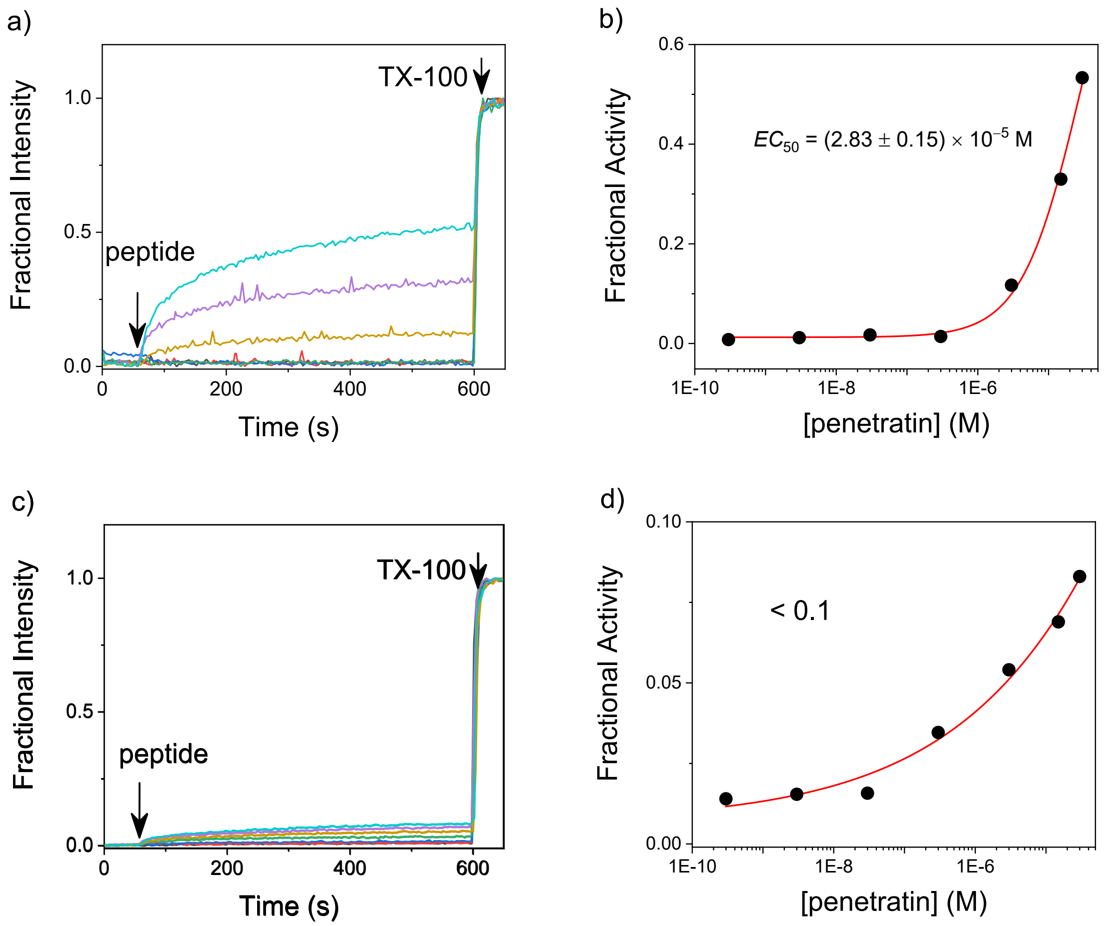

Figure S38. Dual-channel assay with penetratin with POPC⊃CX4/LCG- and POPC⊃CF-LUVs (12.5 μM each) in 10 mM Hepes, 175 mM glucose, pH 7.5. a) Change in fractional emission intensity of the CX4/LCG channel (λ_{ex} = 369 nm; λ_{em} = 475 nm) and b) respective plot of fractional activity at 590 s with varying concentrations of penetratin. c) Change in fractional emission intensity of the CF channel (λ_{ex} = 525 nm: λ_{em} = 545 nm) and d) respective plot of fractional activity at 590 s with varying concentrations of penetratin. In each experiment, varying concentrations of penetratin were added after 60 s and 20 μL of 1.6% TX-100 with 2 mM spermine for calibration after 600 s.

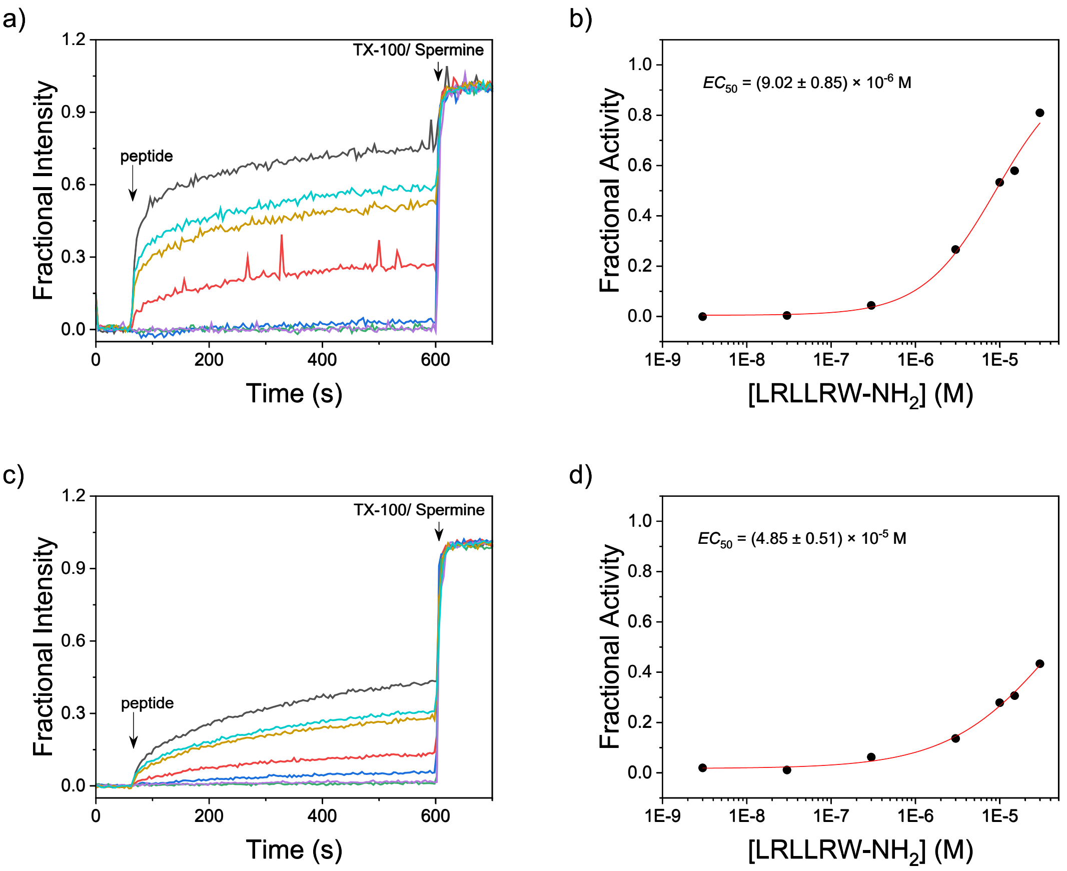

Figure S39. Dual-channel assay with LRLLRW with POPC⊃CX4/LCG- and POPC⊃CF-LUVs (12.5 µM each) in 10 mM Hepes, 175 mM glucose, pH 7.5. a) Change in fractional emission intensity of the CX4/LCG channel (λ_{ex} = 369 nm; λ_{em} = 475 nm) and b) respective plot of fractional activity at 590 s with varying concentrations of LRLLRW. c) Change in fractional emission intensity of the CF channel (λ_{ex} = 525 nm: λ_{em} = 545 nm) and d) respective plot of fractional activity at 590 s with varying concentrations of LRLLRW. In each experiment, varying concentrations of LRLLRW were added after 60 s and 20 µL of 1.6% TX-100 with 2 mM spermine for calibration after 600 s.

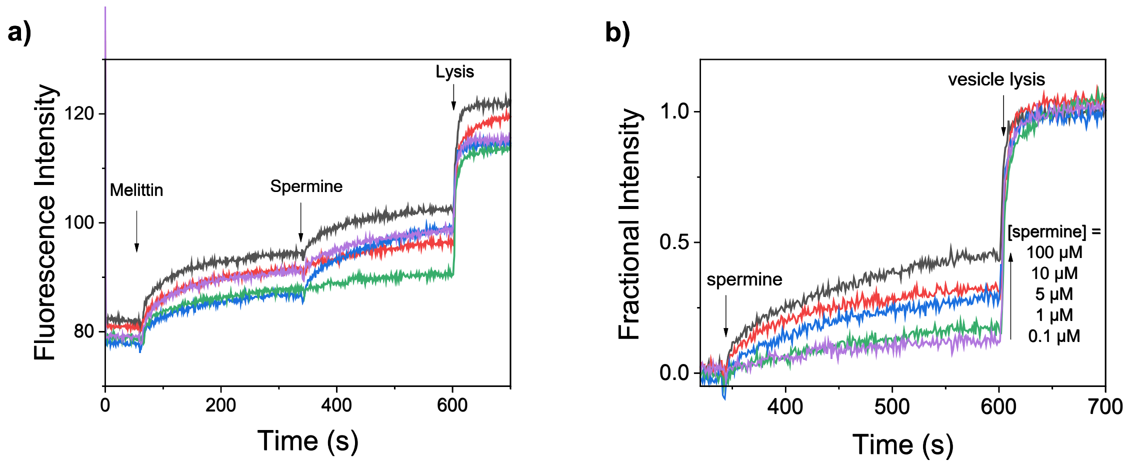

Figure S40. Concentration dependence of subsequently added spermine in a CX4/LCG assay with melittin. The assay was performed with POPC⊃CX4/LCG-LUVs in 10 mM Hepes, 175 mM glucose, pH 7.5 during addition of 3.75 nM melittin at 60 s, varying concentrations of spermine at 340 s, and calibration cocktail at 600 s. a) Original changes in fluorescence intensity of LCG (λ_{ex} = 369 nm, λ_{em} = 502 nm). b) Fractional intensity normalized to 15 s before addition of spermine and after calibration.

Table S10. Single-factor ANOVA for different substances.[a]

Substance	Source	SS	df	MS	*F* value	*p* value	Decision at $p < 0.001$
Penetratin	Between groups	0.3067	1	0.3069	164.58	2.29×10^{-8}	Significant
	Within groups	0.0224	12	0.0019			
	Total	0.3292	13				
Pep-1	Between groups	0.2455	1	0.2455	100.04	3.57×10^{-7}	Significant
	Within groups	0.0295	12	0.0025			
	Total	0.2750	13				
LRLLRW	Between groups	0.2341	1	0.2341	68.53	2.65×10^{-6}	Significant
	Within groups	0.0410	12	0.0034			
	Total	0.2751	13				
TP10	Between groups	0.2778	1	0.2778	101.94	3.23×10^{-7}	Significant
	Within groups	0.0327	12	0.0027			
	Total	0.3105	13				

[a] SS = Sum of Squares, df = degrees of freedom, MS = Mean Square, *F* value = Fisher value (i.e. the between-group variance divided by the within-group variance), *p* value = probability value. The experimental data were obtained by dual-channel measurements using seven independently prepared POPC⊃CX4/LCG-LUV (n = 7) and POPC⊃CF-LUV (n = 7) preparations diluted in 10 mM Hepes, 175 mM glucose, pH 7.5. Peptide concentrations were: Penetratin (15 µM), Pep-1 (10 µM), LRLLRW (30 µM), and TP10 (0.15 µM).

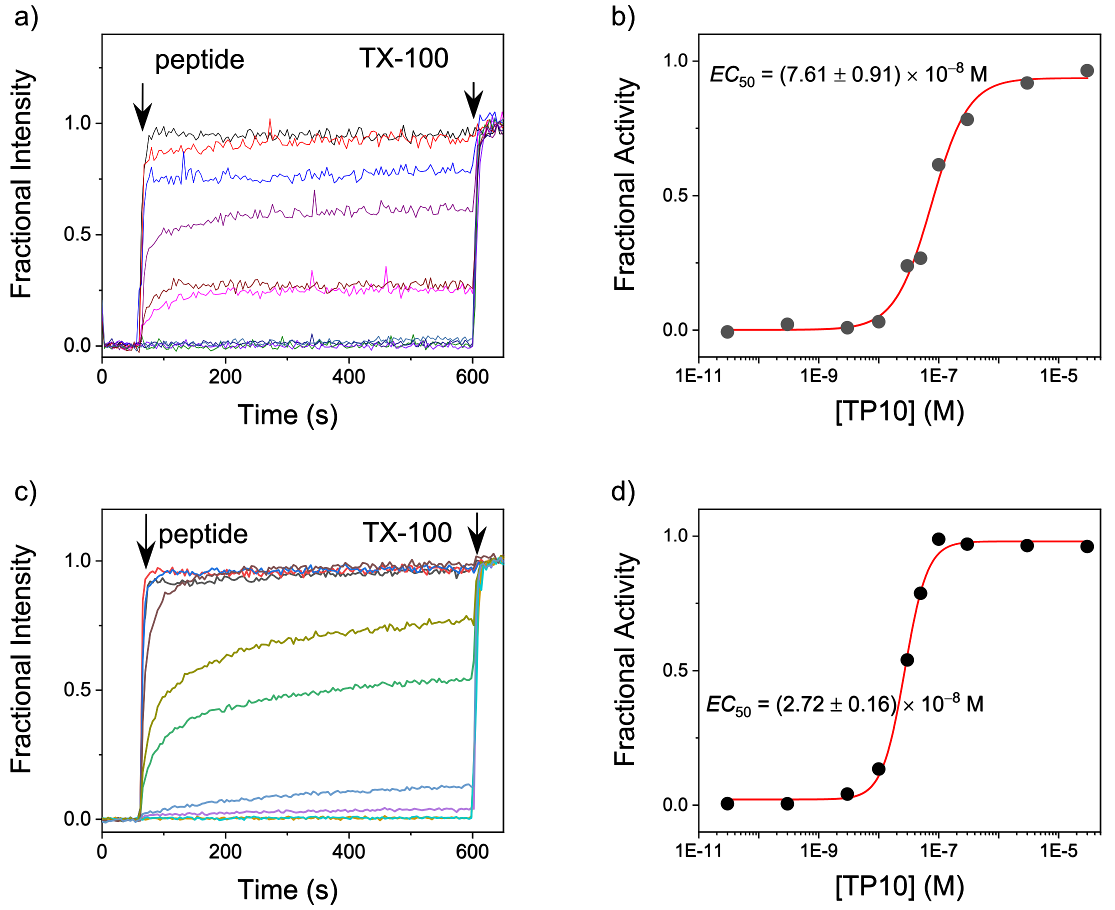

Figure S41. Dual-channel assay with TP10 with POPC/POPS⊃CX4/LCG- and POPC/POPS⊃CF-LUVs (12.5 µM each) in 10 mM Hepes, 175 mM glucose, pH 7.5. a) Change in fractional emission intensity of the CX4/LCG channel (λ_{ex} = 369 nm; λ_{em} = 475 nm) and b) respective plot of fractional activity at 590 s with varying concentrations of TP10. c) Change in fractional emission intensity of the CF channel (λ_{ex} = 525 nm: λ_{em} = 545 nm) and d) respective plot of fractional activity at 590 s with varying concentrations of TP10. In each experiment, varying concentrations of TP10 were added after 60 s and 20 µL of 1.6% TX-100 with 2 mM spermine for calibration after 600 s.

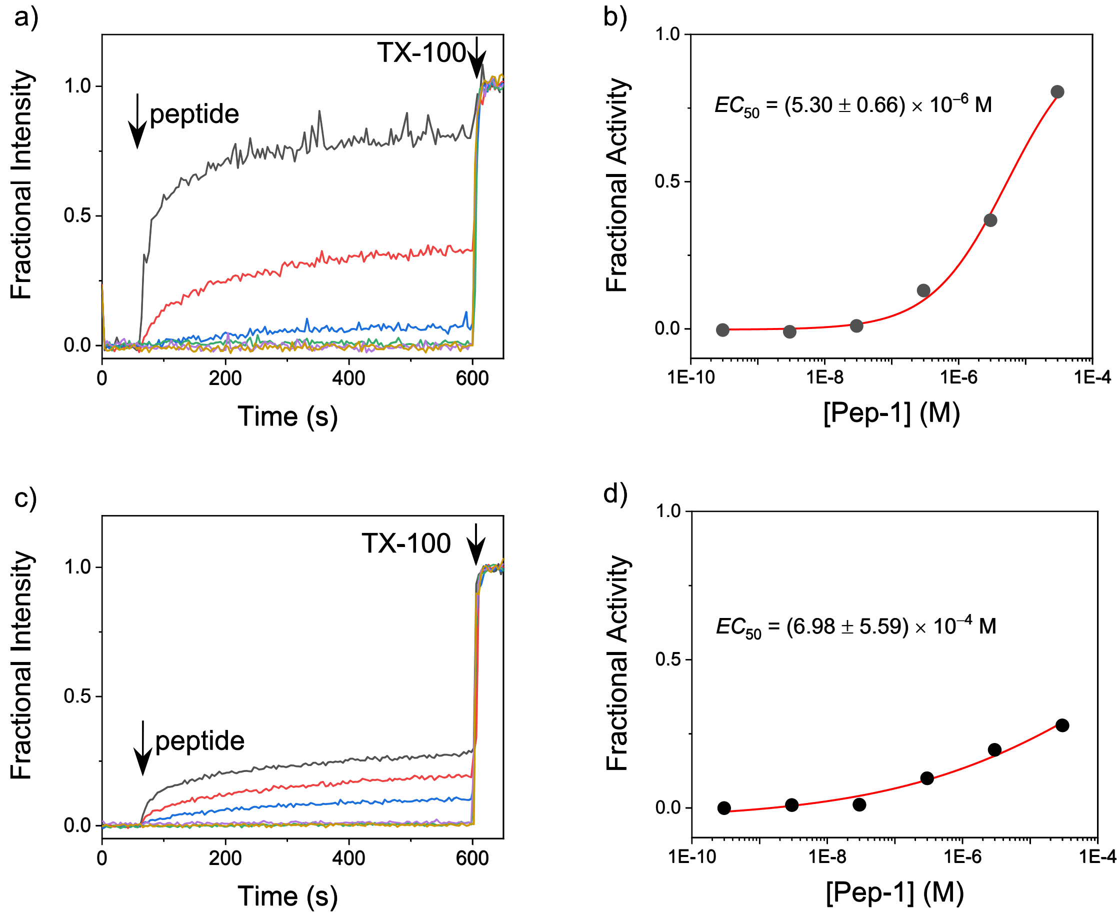

Figure S42. Dual-channel assay with Pep-1 with POPC/POPS⊃CX4/LCG- and POPC/POPS⊃CF-LUVs (12.5 µM each) in 10 mM Hepes, 175 mM glucose, pH 7.5. a) Change in fractional emission intensity of the CX4/LCG channel (λ_{ex} = 369 nm; λ_{em} = 475 nm) and b) respective plot of fractional activity at 590 s with varying concentrations of Pep-1. c) Change in fractional emission intensity of the CF channel (λ_{ex} = 525 nm: λ_{em} = 545 nm) and d) respective plot of fractional activity at 590 s with varying concentrations of Pep-1. In each experiment, varying concentrations of Pep-1 were added after 60 s and 20 µL of 1.6% TX-100 with 2 mM spermine for calibration after 600 s.

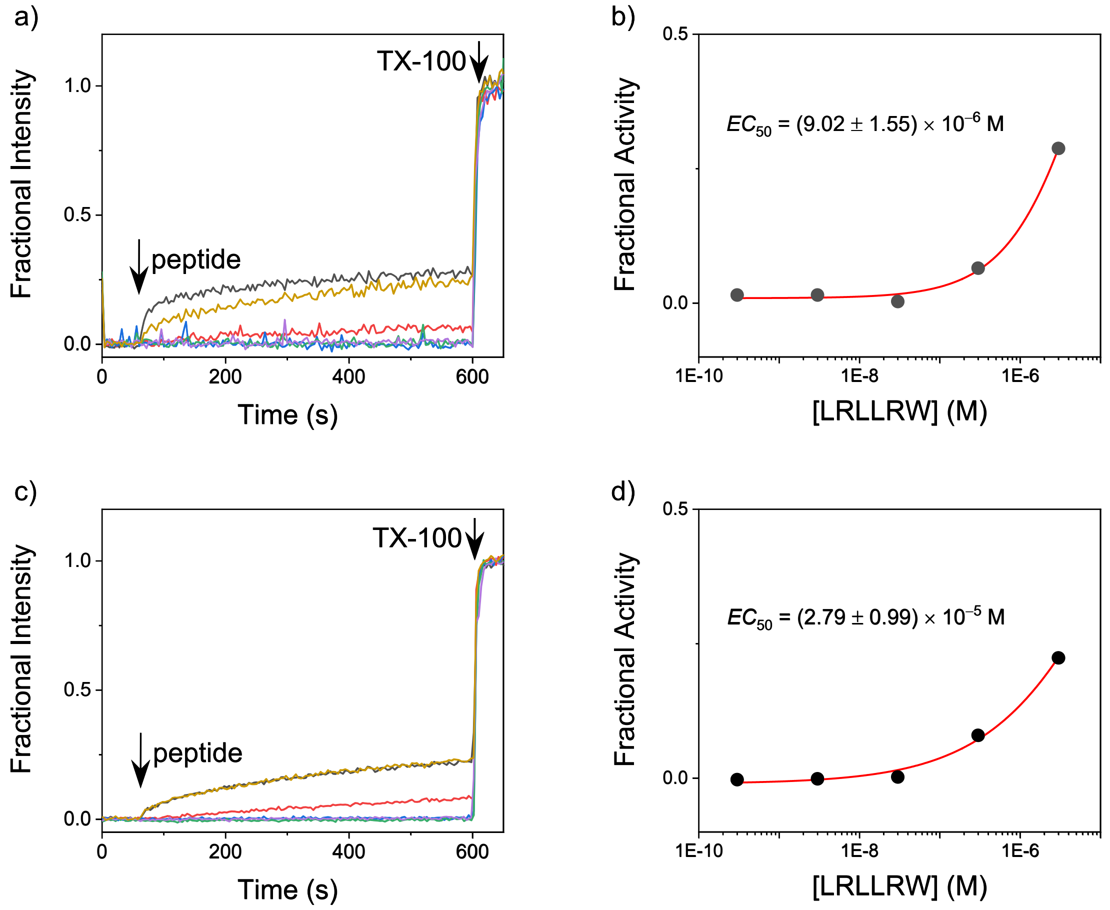

Figure S43. Dual-channel assay with LRLLRW with POPC/POPS⊃CX4/LCG- and POPC/POPS⊃CF-LUVs (12.5 µM each) in 10 mM Hepes, 175 mM glucose, pH 7.5. a) Change in fractional emission intensity of the CX4/LCG channel (λ_{ex} = 369 nm; λ_{em} = 475 nm) and b) respective plot of fractional activity at 590 s with varying concentrations of LRLLRW. c) Change in fractional emission intensity of the CF channel (λ_{ex} = 525 nm: λ_{em} = 545 nm) and d) respective plot of fractional activity at 590 s with varying concentrations of LRLLRW. In each experiment, varying concentrations of LRLLRW were added after 60 s and 20 µL of 1.6% TX-100 with 2 mM spermine for calibration after 600 s.

Table S11. Membrane activity of peptides determined by the dual-channel assay.[a]

Peptide	EC_{50} (µM)	
	CX4/LCG channel	CF channel
TP10	0.076 ± 0.010	0.027 ± 0.002
Pep-1	5.30 ± 0.66	698 ± 55
LRLLRW	9.0 ± 1.6	28 ± 10

[a] Measured with the dual-channel assay with 12.5 µM POPC/POPS(9:1)⊃CX4/LCG-LUVs and 12.5 µM POPC/POPS(9:1)⊃CF-LUVs in 10 mM Hepes, 175 mM glucose, pH 7.5. Errors correspond to the standard deviation obtained by nonlinear fitting (n = 1).

References

[1] H. Mach, C. R. Middaugh, R. V. Lewis, *Anal. Biochem.* **1992**, *200*, 74-80.

[2] B. J. H. Kuipers, H. Gruppen, *J. Agric. Food Chem.* **2007**, *55*, 5445-5451.

[3] A. Hennig, A. Hoffmann, H. Borcherding, T. Thiele, U. Schedler, U. Resch-Genger, *Chem. Commun.* **2011**, *47*, 7842-7844.

[4] a) A. Barba-Bon, Y.-C. Pan, F. Biedermann, D.-S. Guo, W. M. Nau, A. Hennig, *J. Am. Chem. Soc.* **2019**, *141*, 20137-20145; b) S. Peng, A. Barba-Bon, Y.-C. Pan, W. M. Nau, D.-S. Guo, A. Hennig, *Angew. Chem. Int. Ed.* **2017**, *56*, 15742-15745.

[5] R. Hein, C. B. Uzundal, A. Hennig, *Org. Biomol. Chem.* **2016**, *14*, 2182-2185.
